# Interpretable hybrid ensemble with attention-based fusion and EAOO-GA optimization for lung cancer detection

**DOI:** 10.1038/s41598-026-37187-6

**Published:** 2026-03-03

**Authors:** Mesfer Al Duhayyim, Murdhy A. Aldawsari, Atef Ismail, Marwa M. Emam

**Affiliations:** 1 Department of Computer Science, College of Computer Engineering and Sciences, Prince Sattam bin Abdulaziz University, Al-Kharj, 16273 Saudi Arabia; 2 Nursing Department, College of Applied Medical Sciences, Prince Sattam bin Abdulaziz University, Wade Aldwaser, Saudi Arabia; 3 Physics Department, Al-Azhar University, Asyut, 71524 Egypt; 4 Faculty of Computers and Information, Minia University, Minia, Egypt

**Keywords:** Lung cancer, Ensemble learning, Squeeze-and-Excitation networks, Deep learning, Oat optimization algorithm, Cancer, Computational biology and bioinformatics, Mathematics and computing

## Abstract

Lung cancer’s high mortality rate underscores the critical need for early and accurate diagnosis, as late-stage diagnoses often lead to 5-year survival rates as low as 5% compared to 56% for early detection, imposing significant economic burdens on healthcare systems and diminishing patient quality of life. While deep learning models offer promising tools for analyzing Computed Tomography (CT) scans, they often suffer from limitations in generalizability, interpretability, and sensitivity to imbalanced data. This paper introduces SE-FusionEAOO Ensemble, a new robust framework for lung cancer classification. Our approach leverages the strengths of multiple deep learning architectures through a sophisticated two-stage process. First, we construct three powerful feature fusion models by strategically pairing diverse pre-trained networks (DenseNet201/EfficientNetB6, Inception v3/MobileNetV2, DenseNet121/ResNet50), each integrated with Squeeze-and-Excitation (SE) blocks for adaptive feature recalibration. Second, we amalgamate the predictions of these expert models using an intelligently weighted aggregation scheme. The key innovation of our framework is the deployment of a new metaheuristic, the Enhanced Animated Oat Optimization algorithm with Genetic Operators (EAOO-GA), to precisely optimize these ensemble weights, ensuring optimal contribution from each model. To address class imbalance in the IQ-OTH/NCCD lung cancer dataset, we employ the Synthetic Minority Over-sampling Technique (SMOTE), significantly improving the model’s sensitivity to minority classes. Extensive experimental results demonstrate that our framework achieves a state-of-the-art accuracy of 99.40%, with 99.2% precision, 99.5% recall, and 99.3% F1-score, outperforming individual models, conventional ensemble methods, and other metaheuristic optimizers. Additionally, the model was externally validated on the LIDC-IDRI dataset, achieving 97.9% accuracy and 97.8% F1-score, confirming its strong generalization capability across independent clinical domains. The proposed framework provides a highly accurate, reliable, and interpretable tool for automated lung cancer detection.

## Introduction

Lung cancer remains one of the most formidable and pervasive oncological challenges worldwide, constituting a leading cause of cancer-related mortality and presenting a critical public health burden. Its significance is underscored not only by its high incidence and mortality rates but also by its profound impact on patient quality of life and healthcare infrastructure. A primary obstacle in mitigating this disease is the prevalence of late-stage diagnosis^1^. Early-stage lung cancer is often asymptomatic or presents with non-specific symptoms, leading to a substantial proportion of cases being detected only at advanced stages. This diagnostic delay severely constrains treatment efficacy and adversely affects survival outcomes. For instance, while the 5-year survival rate for early-stage detection can be as high as 56%, it plummets to approximately 5% for advanced-stage diagnoses^2^.

The ramifications of lung cancer extend beyond survival statistics, imposing a considerable economic burden on healthcare systems and exacting a heavy physical and emotional toll on patients and their families. Treatment modalities, including surgery, chemotherapy, and radiation therapy, are not only costly but also associated with significant morbidity, further diminishing quality of life^3^. Consequently, there is an urgent and pressing need for innovative diagnostic strategies that enable earlier, more accurate, and cost-effective detection of lung cancer.

From a clinical perspective, lung cancer is broadly categorized into two main histological types: small cell lung cancer (SCLC) and non-small cell lung cancer (NSCLC)^4^. This distinction is crucial for patient management, as each type requires a unique treatment strategy and prognostic assessment. Traditionally, the classification of lung cancer has relied on conventional wet-lab methods and the visual interpretation of medical imaging data, such as computed tomography (CT) scans. Radiologists assess features like tumor size, shape, texture, and location to determine the type and stage of cancer^5^. However, this process is inherently subjective, leading to significant inter-observer variability and potential diagnostic inconsistencies.

The integration of artificial intelligence (AI) and deep learning (DL) into radiology has emerged as a transformative approach to overcome these limitations. AI-driven computational methods offer a powerful solution for automating the analysis of medical images, enabling more objective, precise, and efficient lung cancer classification^6^. These automated systems are particularly vital for the early detection and characterization of pulmonary nodules, distinguishing between benign and malignant manifestations, which is a cornerstone for effective treatment planning and significantly improved patient outcomes^7^. Among these, Convolutional Neural Networks (CNNs) have demonstrated remarkable success, particularly in the domain of image processing and medical image analysis, establishing themselves as the cornerstone of modern computational radiology^8^. This paradigm shift is fundamentally transforming the field of medical image analysis. By leveraging large-scale datasets and sophisticated architectures, DL techniques can extract intricate features from medical images that often elude human perception. This capability has catalyzed substantial improvements in diagnostic accuracy, significantly accelerated analysis times, and reduced the potential for human error. Consequently, AI-based approaches now hold significant potential for the early diagnosis and classification of complex diseases, with lung cancer standing as a primary beneficiary^9^. The automated, precise analysis of medical imaging modalities, such as CT, Magnetic Resonance Imaging (MRI), and histopathology, is pivotal for expediting the diagnostic process and improving patient outcomes^10^.

Consequently, DL is positioned not merely as an assistive tool but as a foundational technology reshaping diagnostic paradigms. The success of CNNs extends beyond lung cancer, with significant achievements across diverse medical imaging modalities, including MRI, histopathology, and mammography. Their integration into Computer-Aided Diagnosis (CAD) systems has proven effective for various conditions, leading to more robust tools that assist clinicians in interpreting complex medical images. A pivotal technique enabling this progress, especially given the challenges of limited annotated medical data, is Transfer Learning (TL)^11^. TL mitigates data scarcity and intricate labeling requirements by adapting CNNs pre-trained on large-scale datasets, such as ImageNet, to specialized medical contexts. This process of knowledge transfer from general to specific domains has been instrumental in enhancing model performance and facilitating the development of accurate, efficient, and scalable diagnostic tools for various types of cancer. By leveraging CNNs pre-trained on large-scale natural image datasets such as ImageNet, transfer learning effectively bridges the domain gap and significantly enhances the performance of models designed for lung cancer detection and classification. This technique facilitates the transfer of generalized feature extraction capabilities, learned from millions of natural images, to the highly specialized domain of thoracic CT analysis. This knowledge transfer is crucial for developing accurate and efficient diagnostic tools, as it mitigates the data scarcity problem inherent in medical imaging and allows models to achieve robust performance even with limited annotated lung CT scans.

Metaheuristic algorithms offer robust and flexible optimization frameworks that can navigate complex search spaces, where traditional methods often falter. Their efficacy has been demonstrated across a vast array of disciplines, including medical image analysis^12–14^ and traffic signal control^15^. To ensure the scientific rigor and performance of our proposed framework, we employ a new proposed metaheuristic, the Enhanced Animated Oat Optimization algorithm with Genetic Operators (EAOO-GA), for critical optimization tasks. This advanced algorithm is utilized to fine-tune model parameters and, most importantly, to derive the optimal weighting scheme for our ensemble fusion, thereby maximizing diagnostic accuracy. Our framework’s performance is rigorously evaluated on a benchmark lung cancer dataset and compared against state-of-the-art methods, demonstrating its significant potential to advance the field of automated medical diagnostics.

In this paper, we introduce an innovative hybrid ensemble framework, the EAOO-Optimized Ensemble, specifically implemented for the accurate and robust detection of lung cancer from CT scans. Moving beyond conventional ensembles that aggregate single models, we leverage the superior representational power of feature fusion architectures. We meticulously designed three distinct fusion pairs: (1) DenseNet201 + EfficientNetB6, (2) Inception v3 + MobileNetV2, and (3) DenseNet121 + ResNet50, selected from a rigorous evaluation of eight top-performing pre-trained models to ensure maximum architectural diversity and complementary feature extraction. Each fusion is further enhanced with Squeeze-and-Excitation (SE) blocks to adaptively recalibrate features and emphasize the most discriminative patterns indicative of malignancy. The key innovation is a cost-effective method that utilizes heat from the breast surface to detect the Enhanced Animated Oat Optimization algorithm with Genetic Operators (EAOO-GA), which intelligently aggregates predictions from these fusion models. This algorithm performs a global search to fine-tune and derive the optimal weighting scheme for the ensemble, ensuring that the most accurate and informative models contribute most significantly to the final diagnosis. This two-stage approach constructs powerful SE, enhanced fusion base learners, and then optimizes their aggregation with EAOO-GA, ensuring superior performance, enhanced robustness, and reliable diagnostic capabilities, directly addressing the critical need for accuracy in medical applications where errors can have severe consequences.

To ensure a rigorous and comprehensive evaluation, the proposed hybrid model is validated using two public benchmark datasets: the Chest CT-Scan dataset and the IQ-OTH/NCCD lung cancer dataset. These datasets provide comprehensive annotations of both cancerous and non-cancerous nodules, enabling a robust assessment of the model’s diagnostic capabilities. A crucial pre-processing pipeline was employed to enhance image quality, standardize inputs, and augment the data, thereby ensuring robust detection accuracy and improving model generalization. Furthermore, to directly combat the pervasive issue of class imbalance common in medical data, the Synthetic Minority Over-sampling Technique (SMOTE) was applied. This critical step ensures the model is not biased toward the majority class and enhances its sensitivity towards detecting less frequent but critical malignant conditions. Furthermore, to visually interpret the decision-making process of our ensemble framework and understand how it focuses on identifying malignant regions within lung CT scans, we employ Gradient-weighted Class Activation Mapping (Grad-CAM) to generate insightful heatmaps. These heatmaps highlight the critical image regions that most significantly influenced the model’s prediction, providing a layer of transparency.

### Problem statement

The application of deep learning to lung cancer detection, while promising, is fundamentally constrained by three persistent challenges: (1) limited model generalizability due to overfitting on training data specifics, (2) high sensitivity to data variability across imaging modalities and acquisition protocols, and (3) a lack of model interpretability, which hinders clinical trust and adoption. While ensemble methods have been employed to improve robustness, they often introduce complexity that exacerbates overfitting and remain a black box. Consequently, there is a critical need for a sophisticated framework that not only enhances accuracy and early detection performance but also explicitly addresses these limitations of generalization, variability, and interpretability through a principled and optimized approach. The proposed SE-FusionEAOO Ensemble directly tackles these issues by: (i) fusing diverse pre-trained models with Squeeze-and-Excitation (SE) blocks for adaptive feature recalibration and resilience to variability, (ii) employing the Enhanced Animated Oat Optimization algorithm with Genetic Operators (EAOO-GA) for precise ensemble weighting to mitigate overfitting and enhance generalization, and (iii) incorporating SMOTE for improved sensitivity to imbalanced classes alongside Grad-CAM for visual interpretability.

### Research motivation

While deep learning has achieved state-of-the-art performance in lung cancer classification, a nuanced yet critical challenge often remains unaddressed: memory overfitting. This phenomenon occurs when powerful models, particularly complex ensembles, merely memorize the statistical noise and specific details of the training data, rather than learning to generalize the underlying diagnostic features. Common regularization techniques, such as dropout and data augmentation, offer partial solutions but are often insufficient to mitigate this subtle form of overfitting, particularly in high-dimensional medical imaging data, as seen in prior CNN and ensemble approaches (e.g., high complexity with limited cross-dataset reliability in^16,17^). Additionally, many methods exhibit sensitivity to data variability (e.g., differences in CT acquisition protocols) and class imbalance prevalent in datasets like IQ-OTH/NCCD, leading to biased predictions and reduced sensitivity for malignant cases^6^. Interpretability remains a significant gap, with most ensembles functioning as “black boxes” despite their strong performance^18^. This gap highlights the urgent need for innovative architectural designs and optimization strategies specifically tailored to counteract memory overfitting, enhance feature robustness, and ultimately ensure that models perform reliably on unseen clinical data, where diagnostic accuracy is crucial. Our framework addresses these challenges by integrating attention-based fusion (SE blocks) for focused, recalibrated features; metaheuristic-driven weighting (EAOO-GA) for optimal aggregation beyond uniform ensembles; SMOTE for balanced training; and Grad-CAM for visual explanations, thereby achieving superior generalization, robustness, and transparency compared to baselines.

### Contribution

The following is a summary of this work’s main contributions, which advance beyond existing methods by incorporating attention-based fusion and metaheuristic optimization for enhanced interpretability and generalization:

- Development of the SE-FusionEAOO Ensemble framework: A novel hybrid architecture that integrates SE blocks into strategic feature fusion pairs of diverse pre-trained networks (DenseNet201/EfficientNetB6, Inception v3/MobileNetV2, DenseNet121/ResNet50). This design enhances feature representation, improves resilience to data variability across imaging protocols, and emphasizes discriminative patterns critical for malignancy detection, outperforming single-model and conventional fusion approaches by adaptive channel-wise recalibration.
- Introduction of an advanced metaheuristic-driven aggregation strategy: Utilization of the novel Enhanced Animated Oat Optimization algorithm with Genetic Operators (EAOO-GA) to optimally determine ensemble weights. This global optimization extends beyond standard methods, precisely balancing contributions from each fusion model to significantly reduce overfitting and boost generalization on unseen data.
- An inherently interpretable diagnostic framework that provides transparency through two mechanisms: SE-based channel-wise feature recalibration and EAOO-optimized weight assignment.
- Comprehensive empirical validation on the IQ-OTH/NCCD lung cancer dataset demonstrating state-of-the-art performance. The proposed framework achieves superior accuracy (99.40%), robustness to data variability, and reduced overfitting compared to individual models, conventional ensemble methods, and other metaheuristic optimization approaches.

### Paper organization

The organization of this paper is as follows: Section 2 offers a brief review of recent studies related to Lung cancer detection and classification. Section 3 provides an overview of the dataset description, architectural components, fusion model architecture, the proposed enhanced optimization algorithm, and the proposed EAOO-GA-Optimized Ensemble framework for lung cancer classification. Moreover, Experimental findings and corresponding analyses are presented in Section 4. Section 5 presented the Advantages, Limitations, and Future Research Directions. Finally, Section 6 outlines the concluding remarks and potential directions for future research.

## Literature review

Medical image analysis for pulmonary oncology has emerged as a critical research domain, driving innovation in computational diagnostics. This section surveys contemporary methodologies developed to augment the precision and robustness of automated lung cancer detection systems, critically examining their technical foundations, strengths, and inherent constraints. We organize the related studies into subsections focusing on deep learning models, ensemble and fusion methods, and metaheuristic optimization techniques, before discussing persistent limitations and gaps.

### Deep learning models for lung cancer detection

Mohamed et al.^19^ proposed a hybrid model that combines CNNs with the Ebola Optimization Search Algorithm (EOSA) to enhance lung cancer classification in CT images. The approach aimed to optimize CNN weights and biases using EOSA, addressing challenges in CNN configuration. Evaluated on the IQ-OTH/NCCD dataset, the EOSA-CNN model achieved an accuracy of 93.21% and demonstrated superior performance compared to other metaheuristic-CNN methods, particularly in classifying normal and malignant cases. In addition, Eram et al.^20^ introduced an improved DenseNet201 model that combines transfer learning with explainable artificial intelligence to classify various lung diseases from X-ray images. Its performance was evaluated against other transfer learning models, including EfficientNetB0, InceptionV3, and LeNet, using standard evaluation metrics. Imran et al.^21^ proposed a transformer-based hierarchical model for non-small cell lung cancer (NSCLC) detection and classification from histopathological images. By integrating CNNs for local feature extraction and vision transformers (ViTs) for capturing long-range dependencies, the model classifies NSCLC into normal, adenocarcinoma, and squamous cell carcinoma categories. Evaluated on the LC25000 dataset, it achieved an accuracy of 98.8%, outperforming state-of-the-art methods in precision and recall. Sewatkar^22^ introduced the MeVs-deep CNN, an optimized deep learning model for efficient lung cancer classification using PET/CT images. The approach employs Memory-Enabled Vulture Search Optimization to segment and classify data after preprocessing with Non-Local Means filtering, demonstrating improved autonomy and accuracy in categorization.

Furthermore, Elkenawy et al.^23^ developed a hybrid framework combining the Greylag Goose Optimization (bGGO) algorithm with a multilayer perceptron for lung cancer classification. By enhancing feature selection and applying comprehensive preprocessing, their method outperformed several binary optimization algorithms and achieved 98.4% accuracy, with results validated through statistical tests and performance analysis. Zhang et al.^1^ introduced a DenseNet-based CNN framework enhanced with data fusion and mobile edge computing for lung cancer classification. The approach leverages preprocessing and multi-source data integration to improve accuracy, while edge computing enables real-time CT scan analysis. The model classifies lung tissue into Normal, Benign, or Malignant, with malignant cases further categorized into adenocarcinoma, squamous cell carcinoma, and large cell carcinoma. Shafi and Chinnappan^24^ presented a hybrid transformer-CNN and LSTM model for lung disease segmentation and classification. The approach includes preprocessing with median filtering, segmentation using an improved Transformer-based CNN (ITCNN), feature extraction (e.g., texture via modified LGIP), and classification with a hybrid LinkNet-Modified LSTM (L-MLSTM), outperforming existing models in accuracy. Lakshminarasimha et al.^25^ developed an optimization-driven deep learning approach for enhancing lung cancer diagnosis from CT images. The model integrates CBAM with EfficientNet for feature extraction and applies optimization algorithms like Gray Wolf Optimization (GWO) for hyperparameter tuning, achieving accuracies of 99.81% and 99.25% on the Lung-PET-CT-Dx and LIDC-IDRI datasets, respectively.

### Ensemble and fusion methods

Moreover, Shariff et al.^26^ introduced a CNN framework enhanced with Differential Augmentation (DA) to mitigate memory overfitting and improve model generalization on unseen lung cancer data. The DA strategy involved applying targeted augmentations, such as hue, brightness, saturation, and contrast adjustments, to increase data diversity and robustness. The model was evaluated on multiple datasets, including IQ-OTH/NCCD, and optimized using Random Search for hyperparameter tuning. The CNN and DA model achieved a classification accuracy of 98.78%, surpassing several state-of-the-art architectures, including DenseNet, ResNet, EfficientNetB0, and ensemble-based approaches. Statistical validation, as confirmed by Tukey’s HSD post-hoc test, demonstrated the significance of the model’s superior performance, highlighting its effectiveness in overcoming overfitting and enhancing cross-dataset reliability.

Pamungkas et al.^27^ proposed a hybrid framework that combines VGG-based CNNs with GAN-driven data augmentation to enhance lung cancer classification. By generating realistic synthetic images for underrepresented classes, their method mitigates class imbalance and enhances model generalization. Validated on the IQ-OTH/NCCD and Lung Cancer CT Scan datasets, the VGG-GAN approach achieved improved performance across both binary and multi-class classifications. In addition, Kumaran et al.^7^ presented an explainable deep learning framework that integrates three pre-trained models: VGG16, ResNet50, and InceptionV3 within a unified ensemble to enhance lung cancer diagnosis from medical images. The approach standardizes input images through resizing and format conversion to maintain consistency across datasets and maximize model performance. Durgam et al.^28^ proposed the Cancer Nexus Synergy (CanNS) framework for enhancing lung cancer detection through integrated deep learning and transformer models. It combines Swin-Transformer UNet (SwiNet) for segmentation, Xception-LSTM GAN (XLG) CancerNet for classification, and Devilish Levy Optimization (DevLO) for parameter fine-tuning, achieving superior accuracy, sensitivity, and specificity compared to prior methods. Gandhi et al.^29^ introduced a Hybrid Attention Vision Transformer (HViT) for enhanced lung cancer detection. The model leverages attention mechanisms to capture complex features in multi-modal clinical images, improving accuracy in early-stage detection and generalizing across diverse datasets via transfer learning, though with potential high computational costs.

### Metaheuristic optimization methods

Malik et al.^30^ developed a model for optimizing chemotherapeutic targets in non-small cell lung cancer using transfer learning for precision medicine. It employs a hybrid UNet transformer for feature extraction, modified Rime optimization (MRO) for dimensionality reduction, and a deep transfer learning (DTransL) model, achieving accuracies up to 98.398% on benchmark datasets like Davis, KIBA, and Binding-DB.

Moreover, some studies have explored multi-objective metaheuristic optimization and ensemble strategies across diverse classification domains. For instance, Dhal *et al.* proposed multi-stage and zone-oriented multi-objective frameworks for multi-label feature selection and classification^31,32^. They also introduced multi-objective deep learning systems for clinical disease prediction and histopathological cancer diagnosis^33,34^. While these works successfully demonstrate the utility of multi-objective optimization in handling complex feature interactions, our approach differs fundamentally by employing the Enhanced Animated Oat Optimization with Genetic Operators (EAOO-GA) to adaptively optimize ensemble weights in a single-label medical imaging context.

Recently, several advanced hybrid metaheuristic algorithms have been introduced to improve optimization performance in both engineering and medical domains. Mahapatra *et al.* proposed the Fast-Flying PSO (FF-PSO)^35^ and Quantized Orthogonal Experimentation SSA (QOX-SSA)^36^, which enhance swarm intelligence and salp swarm optimization by incorporating quantization and orthogonal experimentation strategies to balance global and local search. Their subsequent Adaptive Dimensional Search SSA (ADOX-SSA)^37^ further improved convergence through adaptive search dimension control. Similarly, Agrawal *et al.* introduced Local Search SSA-driven Deep CNN for brain tumor analysis^38^, demonstrating the utility of hybrid swarm optimizers for deep network fine-tuning, and later proposed the Quantum-Inspired Adaptive Mutation PSO (QAMO-PSO)^39^ for robust global search and parameter adaptation. In contrast, our proposed EAOO-GA framework employs a novel bio-inspired Animated Oat Optimization enhanced with genetic operators to adaptively optimize ensemble weights for lung cancer CT image classification. While conceptually related to these hybrid metaheuristics, EAOO-GA uniquely integrates evolutionary and biologically inspired dynamics within an ensemble deep learning context, ensuring both diagnostic interpretability and computational efficiency.

### Limitations and research gaps

Moreover, a comprehensive review of the current literature reveals significant advancements in deep learning for lung cancer diagnosis, primarily through the use of sophisticated CNNs, ensemble methods, and transfer learning. However, as critically summarized in Table 1, these approaches consistently encounter persistent limitations that hinder their clinical deployment. Key among these are a prevalent trade-off between model complexity and interpretability, a high computational burden, especially for ensemble techniques, and challenges in generalizing to diverse, real-world data. Many models also remain vulnerable to data-specific biases and often lack transparency in their decision-making processes. It is these identified gaps, particularly the need for a robust yet interpretable model that generalizes effectively without prohibitive computational cost, that motivate the proposed framework in this study.

**Table 1:** Comparative summary of representative deep learning works for lung cancer diagnosis, including methods, advantages, and disadvantages.

Author	Method	Advantages	Disadvantages
Islam et al.^40^	Review of DL-based data augmentation (e.g., GANs)	Effectively addresses data scarcity and quality issues in medical imaging through advanced augmentation	Increased model complexity and computational cost, potentially reducing interpretability and training efficiency.
Saha et al.^16^	VER-Net (ensemble of three TL models)	Enhances classification accuracy by combining complementary transfer learning models	High structural complexity hinders model interpretability and increases computational demands.
Klén et al.^41^	CNN with various augmentation methods	Provides comprehensive evaluation of augmentation techniques across modalities	Findings are highly data-dependent, with a lack of generalizability across all clinical scenarios and image types.
Kukreja et al.^42^	CNN for histology classification	Targeted multi-class histology classification with focused architecture	Limited comparative analysis; performance not benchmarked against a wider range of state-of-the-art models.
Zhang et al.^1^	CNN-DenseNet fusion with edge computing	Facilitates real-time analysis and high accuracy via data fusion and edge deployment	Potential challenges in generalizing to diverse real-world datasets due to data variability.
Gai et al.^43^	CNN-DenseNet fusion	Strong performance in tissue type discrimination	Similar to^1^, it may face generalization issues on heterogeneous clinical data.
Quasar et al.^18^	Ensemble of BEiT, DenseNet, Sequential CNN	Improves precision by leveraging diverse architectural strengths	The ensemble’s black-box nature reduces interpretability.
Raza et al.^6^	Lung-EffNet (EfficientNet-based)	Efficient design suitable for clinical deployment	Reliance on augmentation may hurt generalization.
Gautam et al.^17^	Ensemble of ResNet-152, DenseNet-169, EfficientNet-B7	Robust performance from strong pre-trained models	High computational cost limits clinical applicability.
Dritsas et al.^44^	Feature ranking with ML and DL	Effective balancing and bootstrapping	Performance is limited by available features.
Tsou et al.^45^	XGBoost on breath VOC data	Non-invasive biomarker exploration	Potential study-design bias.
Shah et al.^46^	Deep Ensemble 2D CNN	High detection accuracy (95%)	Computationally expensive.
Mikhael et al.^47^	Sybil Risk Prediction Model	Personalized screening support	Dataset demographic dependency.
Wankhade et al.^48^	CCDC-HNN (3D CNN)	Captures volumetric features	High memory and computation needs.
Said et al.^49^	UNETR with SSL	Strong transformer-based performance	Requires large data and compute.
Wani et al.^50^	CNN–XGBoost Hybrid	Improved interpretability via SHAP	Added complexity and latency.
Chae et al.^51^	Deep Texture Learning	Clinical insight into ILA	Limited generalizability.
Mohamed et al.^19^	EOSA-CNN	High accuracy via optimization	Long convergence time.
Rajasekar et al.^52^	DL on Histopathology	Early detection capability	Dataset not specified.
Deepapriya et al.^53^	CNNs for X-ray and CT	Fast multi-modal diagnosis	Difficulty with subtle differences.
Shariff et al.^26^	CNN with Differential Augmentation	98.78% accuracy with strong generalization	Hyperparameter sensitivity.
Durgam et al.^28^	Integrated DL-Transformer	Improved accuracy and robustness	High computational demand.
Shafi et al.^24^	Transformer–CNN	Rich feature extraction	Heavy preprocessing required.
Imran et al.^21^	Transformer-CNN Hierarchy	98.8% NSCLC accuracy	Overfitting risk.
Malik et al.^30^	Hybrid UNet Transformer	High precision for precision medicine	Long training time.

## Methodology

**Fig. 1:**
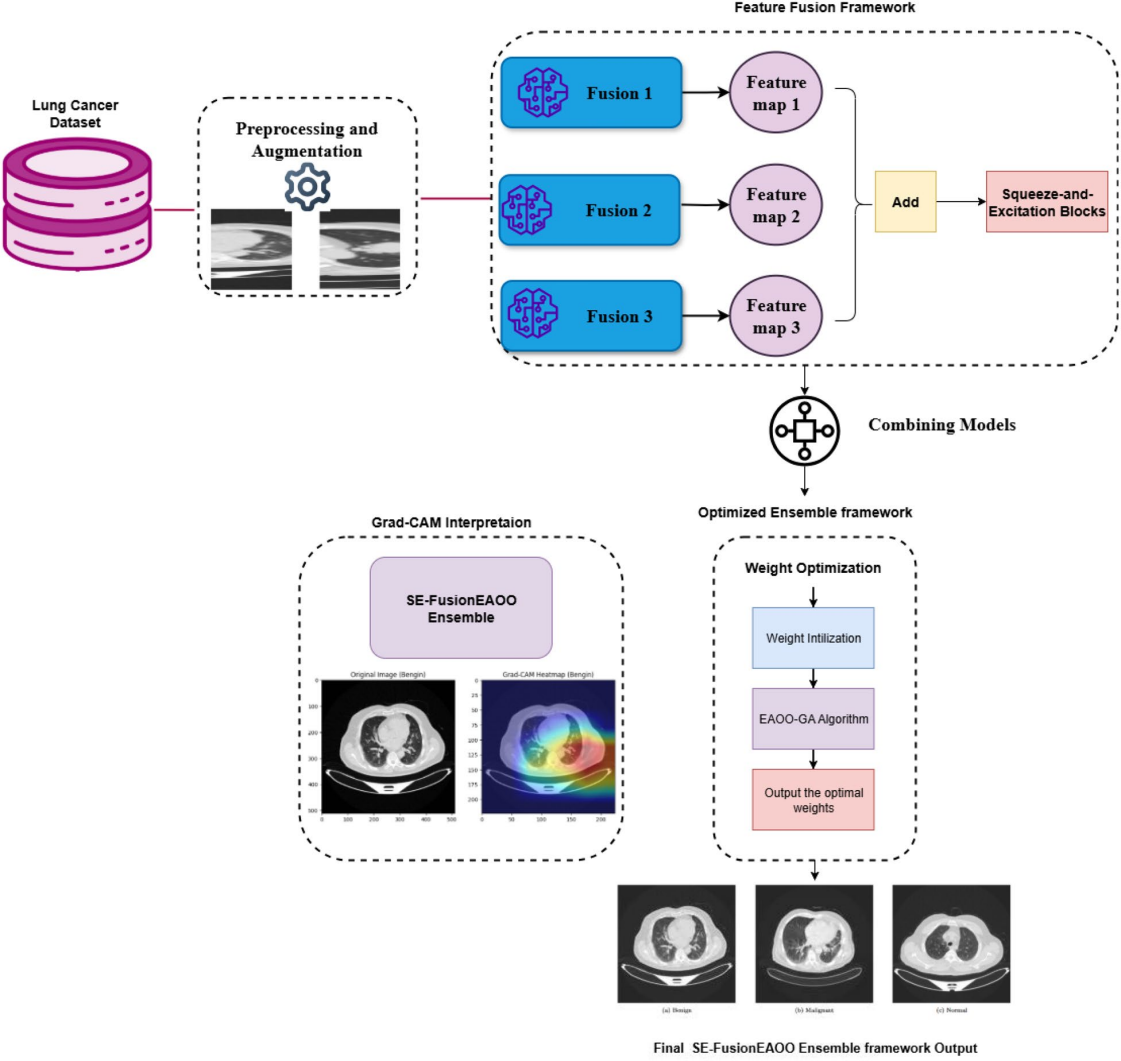
Overall structure of the proposed SE-FusionEAOO Ensemble framework for lung cancer detection.

This study presents the SE-FusionEAOO Ensemble, a multi-stage hybrid framework developed to achieve state-of-the-art accuracy, robustness, and interpretability in lung cancer detection from CT scans. The overall system architecture is depicted in Fig. 1. The proposed methodology proceeds through the following structured stages: (1) Dataset preprocessing, augmentation, and class imbalance mitigation using SMOTE (Section 3.1); (2) Incorporation of Squeeze-and-Excitation (SE) blocks for adaptive channel recalibration (Section 3.2); (3) Rigorous evaluation and selection of six top-performing pre-trained models from eight candidates via transfer learning (Section 3.3); (4) Development and deployment of the proposed Enhanced Animated Oat Optimization with Genetic Operators (EAOO-GA) for optimal ensemble weight determination (Section 3.4, Algorithm 1); (5) Construction of three SE-enhanced fusion architectures by strategically pairing the selected models: DenseNet201 + EfficientNetB6, InceptionV3 + MobileNetV2, and DenseNet121 + ResNet50, each enhanced with SE modules (Section 3.5, Fig. 5, 6); (6) Weighted aggregation of predictions from the three fusion models using EAOO-GA-optimized weights to produce the final classification output (Section 3.6, Algorithm 2); and (7) Integration of **Grad-CAM** for model interpretability and visualization of decision-relevant regions (Section 3.6.2).

### Dataset description and preprocessing

#### Dataset description

**Fig. 2:**
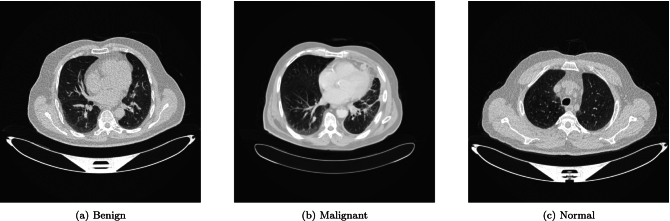
Visual samples from the preprocessed IQ-OTH/NCCD dataset, representing the three distinct classes: (**a**) Benign, (**b**) Malignant, and (**c**) Normal. All images have been standardized to a resolution of $256 \times 256$ pixels.

This paper employs the publicly available IQ-OTH/NCCD lung cancer dataset^54^, a well-established benchmark for the development of CAD systems. The dataset comprises CT scan images categorized into three critical classes: Benign, Malignant, and Normal, as shown in Fig. 2. A significant challenge posed by this dataset is the inherent heterogeneity in image dimensions. A detailed breakdown of the image size distribution per class, provided in Table 2, reveals this complexity. While the majority of images ($512 \times 512$ pixels) form a homogeneous subset, there are notable exceptions that necessitate meticulous preprocessing. Specifically, the Malignant class contains images sized $512 \times 623$ and $512 \times 801$, and a single image of $404 \times 511$. The Normal class also contains a single outlier sized $331 \times 506$. This precise understanding of the data structure is crucial for designing tailored preprocessing steps to ensure spatial uniformity for model input. Resizing and standardization strategies must account for these variations to prevent the loss of critical diagnostic information or the introduction of distortions.

The overall class distribution is also a key consideration. As shown in Table 2, the Malignant class is the most populous, followed by Normal and then Benign cases. This imbalance is a common characteristic of medical imaging datasets and must be addressed during model training and evaluation to prevent algorithmic bias towards the majority class.

**Table 2:** Detailed class and image size distribution of the IQ-OTH/NCCD dataset.

Class	Image Size	Number of Samples
Benign	$512 \times 512$	120
Malignant	$512 \times 512$	501
	$512 \times 623$	31
	$512 \times 801$	28
	$404 \times 511$	1
Normal	$512 \times 512$	415
	$331 \times 506$	1
**Total**		**1097**

The IQ-OTH/NCCD dataset, while a valuable resource, is subject to potential biases common in medical imaging datasets. These biases must be acknowledged as they impact the generalizability of trained models. A primary concern is the limited documentation regarding patient demographics (e.g., age, gender, ethnicity) and the variety of imaging equipment used. A homogeneity in these factors could limit model performance when applied to broader, more diverse populations or data from different scanners. Furthermore, as detailed in Table 2, the dataset exhibits a pronounced class imbalance, characterized by a substantial overrepresentation of Malignant instances relative to Benign cases. This disparity introduces a significant risk of algorithmic bias, predisposing the model to overfit the prevalent majority class and consequently impairing its predictive accuracy for the critically important minority class, a scenario that is untenable in medical diagnostics, where equitable performance across all pathologies is mandatory. To counteract this bias and fortify model generalizability, we implemented a dual strategy: comprehensive data augmentation to increase phenotypic diversity, and the Synthetic Minority Over-sampling Technique (SMOTE) to strategically oversample the Benign class. Furthermore, model validation was conducted on external cohorts to ensure robustness and clinical applicability across diverse patient demographics.

Furthermore, the proposed SE-FusionEAOO framework was performed using the publicly available Lung Image Database Consortium and Image Database Resource Initiative (LIDC-IDRI) dataset^55^. This dataset is widely adopted for developing and benchmarking CAD systems for pulmonary nodule detection and classification. It consists of thoracic CT scans from 1,018 subjects, each annotated by four experienced radiologists who provided nodule-level assessments for lesions with diameters $\ge$ 3 mm. Each annotation includes clinically relevant features such as malignancy likelihood, texture, sphericity, and subtlety, which are strongly correlated with cancer risk. For this study, we excluded nine CT cases exhibiting inconsistent slice spacing and 121 cases with slice thicknesses $\ge$ 3 mm, resulting in a final cohort of 888 valid scans. Nodules were categorized as benign or malignant based on consensus malignancy ratings among the radiologists. The retained scans had slice thicknesses ranging from 0.6 mm to 2.5 mm and in-plane resolutions between 0.48 mm and 0.72 mm. This dataset is widely adopted for developing and benchmarking computer-aided diagnosis (CAD) systems for pulmonary nodule detection and classification. It consists of thoracic CT scans from 1,018 subjects, each annotated by four experienced radiologists who provided nodule-level assessments for lesions with diameters $\ge$ 3 mm. Each annotation includes clinically relevant features such as malignancy likelihood, texture, sphericity, and subtlety, which are strongly correlated with cancer risk. For this study, we excluded nine CT cases exhibiting inconsistent slice spacing and 121 cases with slice thicknesses $\ge$ 3 mm, resulting in a final cohort of 888 valid scans. Nodules were categorized as benign or malignant based on consensus malignancy ratings among the radiologists. The retained scans had slice thicknesses ranging from 0.6 mm to 2.5 mm and in-plane resolutions between 0.48 mm and 0.72 mm. Importantly, the LIDC-IDRI dataset was used for external validation to ensure true out-of-distribution generalization, as model training and optimization were performed exclusively on the IQ-OTH/NCCD dataset.

#### Data preprocessing

**Fig. 3:**
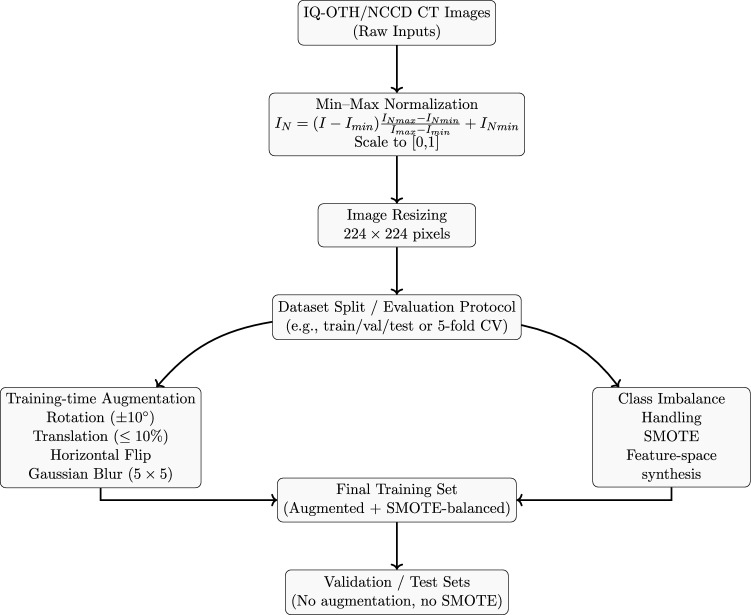
Flowchart of the preprocessing pipeline.

The preprocessing pipeline used in the proposed framework is shown in Fig. 3.

Normalization serves as a vital preprocessing step, primarily for scaling images and standardizing pixel intensities. This standardization promotes stable and efficient model convergence during training. Initially, each input image undergoes intensity normalization, rescaled to a [0, 1] range using min-max normalization, as defined by the following equation^56^:

1 $$\begin{aligned} I_N=(I-I_{min})\times \frac{I_{Nmax}-I_{Nmin}}{I_{max}-I_{min}} + I_{Nmin} \end{aligned}$$

where *I* denotes the input image with pixel values bounded by $I_{\text {max}}$ and $I_{\text {min}}$, and $I_N$ represents the normalized image with values scaled to the range $[I_{N_{\text {min}}}, I_{N_{\text {max}}}]$. Subsequently, each image in the dataset was resized to a spatial resolution of $224 \times 224$ pixels to ensure dimensional consistency across all samples.

#### Data augmentation

To enhance model robustness, mitigate overfitting, and ensure generalization to unseen data, a comprehensive suite of data augmentation techniques was employed. These transformations were carefully selected to mimic real-world variations inherent in medical imaging. Furthermore, the pronounced class imbalance within the dataset was addressed using synthetic oversampling. The augmentation pipeline included the following transformations, applied randomly during training:

- **Rotation:** Images were randomly rotated by angles between $-10$ and 10 degrees to simulate different anatomical orientations encountered during scanning.
  2 $$\begin{aligned} I_{\text {rotated}} = \text {rotate}(I_{\text {original}}, \theta ), \quad -10 \le \theta \le 10 \end{aligned}$$
- **Translation:** Images were shifted horizontally and vertically by up to $10\%$ of their dimensions to account for variations in lung positioning within the scanner’s field of view.
  3 $$\begin{aligned} I_{\text {translated}} = \text {translate}(I_{\text {original}}, d_x, d_y), \quad |d_x|, |d_y| \le 0.1 \times \text {image}\_\text {size} \end{aligned}$$
- **Horizontal Flipping:** Mirror images were generated to introduce natural variability in image presentation, a common occurrence in clinical settings.
  4 $$\begin{aligned} I_{\text {flipped}} = \text {flip}(I_{\text {original}}) \end{aligned}$$
- **Gaussian Blurring:** A $5\times 5$ kernel was applied with an automatically calculated standard deviation to simulate slight focus variations and reduce image noise, thereby conditioning the model to handle practical diagnostic challenges without distorting critical lung tissue details.
  5 $$\begin{aligned} I_{\text {blurred}} = G_{\sigma } * I_{\text {original}} \end{aligned}$$

#### Handling class imbalance

Initial data analysis revealed a significant class imbalance, with ’Malignant’ cases substantially outnumbering ’Benign’ and ’Normal’ cases (Table 3). To prevent model bias towards the majority class, the SMOTE was employed. SMOTE synthesizes new examples in the feature space of the minority classes by interpolating between existing instances, effectively balancing the class distribution and enriching the dataset with diverse examples of underrepresented classes.

**Table 3:** Class distribution before and after applying SMOTE.

Class	Before SMOTE	After SMOTE
Benign	420	420
Malignant	312	420
Normal	90	420

**Fig. 4:**
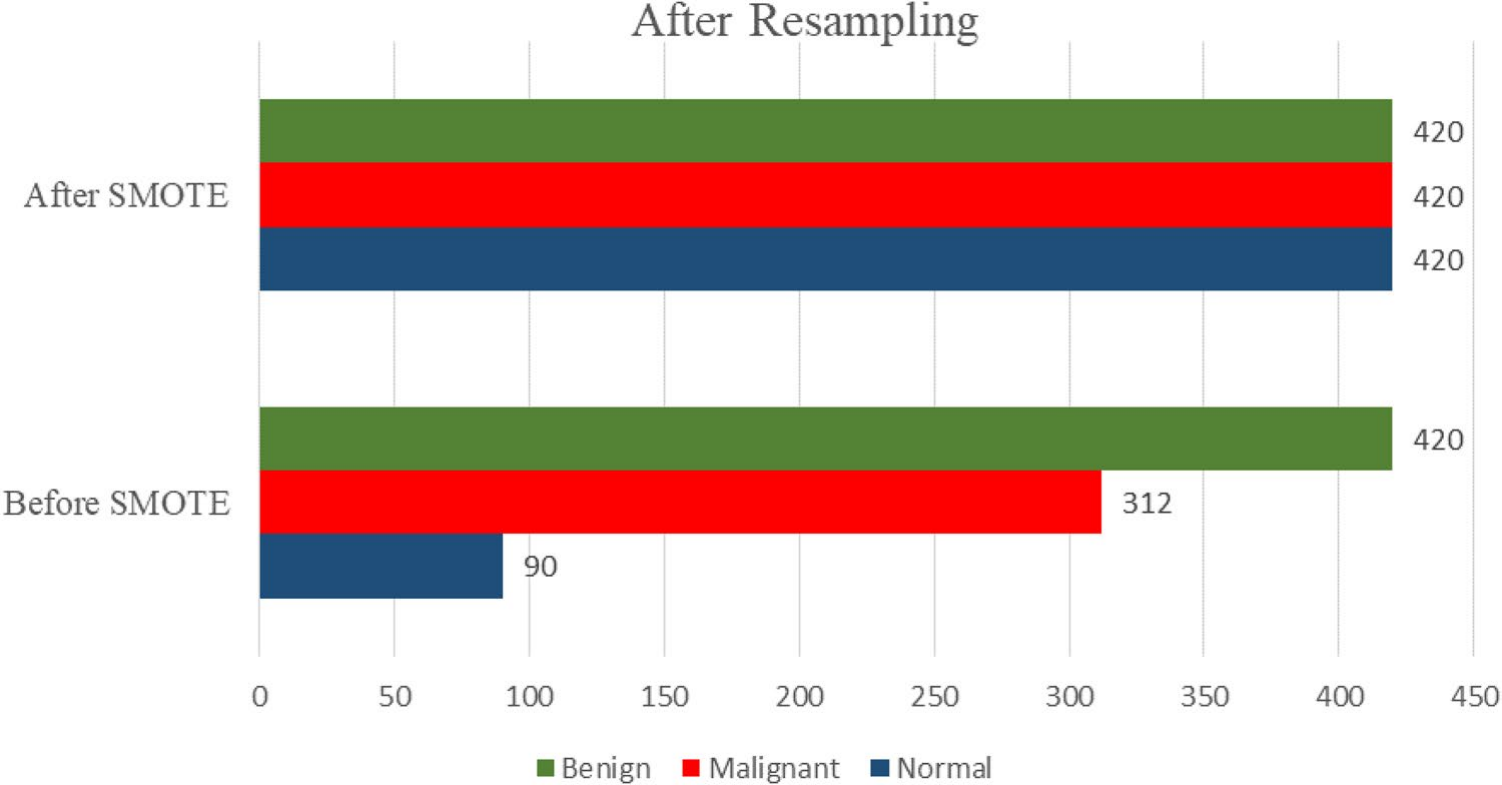
Analysis of class distribution before and after applying the SMOTE.

This balanced distribution, achieved through SMOTE and visualized in Fig. 4, ensures all classes have equal representation during training. This prevention of model bias towards the majority class (‘Malignant’) forces the learning of discriminative features across all categories, Benign, Malignant, and Normal, with improved accuracy. Consequently, this strategy significantly enhances the model’s overall diagnostic reliability and generalizability across all case types.

### Squeeze-and-excitation blocks

To enhance the feature representation and discriminative power of our CNN, we integrated SE blocks, a foundational architectural unit introduced by^57^. These blocks act as a self-attention mechanism that dynamically recalibrates channel-wise feature responses, enabling the network to autonomously emphasize informative features and suppress less relevant or redundant ones. The SE block operates through a sequential two-phase process: squeeze and excitation. The squeeze phase employs global average pooling to aggregate spatial information from each feature map. This operation transforms a feature map tensor $\textbf{U} \in \mathbb {R}^{H \times W \times C}$ into a channel-wise descriptor vector $\textbf{z} \in \mathbb {R}^{C}$, effectively capturing the global distribution of responses for each channel:

6 $$\begin{aligned} z_c = \frac{1}{H \times W} \sum _{i=1}^{H} \sum _{j=1}^{W} u_c(i, j) \end{aligned}$$

Subsequently, the excitation phase processes this descriptor using a simple multi-layer perceptron (MLP), typically comprising two fully connected layers, to model nonlinear interactions and dependencies between channels. This MLP acts as a gating mechanism, generating a vector of adaptive scaling weights $\textbf{s} \in \mathbb {R}^{C}$ where each value is between 0 and 1:

7 $$\begin{aligned} \textbf{s} = \sigma (\textbf{W}_2 \delta (\textbf{W}_1 \textbf{z})) \end{aligned}$$

Where, $\textbf{W}_1$ and $\textbf{W}_2$ are the learned weights of the MLP, $\delta$ denotes the ReLU activation function, and $\sigma$ is the sigmoid activation function that normalizes the output. The final output of the SE block is produced by rescaling the original feature map $\textbf{U}$ channel-wise with the learned weights: $\tilde{\textbf{x}}_c = s_c \cdot \textbf{u}_c$.

To clarify the technical integration: SE blocks are inserted post-feature extraction in each fusion pair, dynamically modeling channel interdependencies to prioritize malignancy-indicative patterns (e.g., nodule texture) while suppressing noise, as formalized in Eqs. (6)-(7). This enhances both accuracy and interpretability by revealing the importance of features.

#### Rationale and integration of SE for lung cancer detection in feature fusion

In the specific context of lung cancer detection from CT scans, this adaptive feature recalibration mechanism is critically advantageous. The network learns to assign higher weights to feature channels that encode semantically salient patterns indicative of malignant nodules, such as spiculation, lobulation, or ground-glass opacity, while attenuating channels associated with normal parenchyma or benign structures. This selective emphasis not only bolsters classification accuracy by focusing on the most discriminative evidence but also inherently improves the model’s interpretability. By examining the excitation weights, one can gain insights into which visual features the model deems most significant for its diagnosis, thereby fostering greater clinical trust and verification. This capability is further amplified by the strategic integration of SE blocks within our feature fusion architecture. Rather than merely concatenating features from different backbone networks, the SE mechanism actively recalibrates the combined feature maps post-fusion. This induces a powerful complementary effect, prioritizing and amplifying the most salient diagnostic features from each network while suppressing redundant or contradictory information. Consequently, the final fused representation is not only comprehensive but also optimally attuned to the specific task of lung nodule classification.

### Deep feature extraction via transfer learning

In the domain of medical image analysis, particularly for lung cancer detection, the scarcity of large, annotated datasets presents a significant challenge. Training CNNs from scratch under such constraints is computationally prohibitive and highly susceptible to overfitting, as it demands substantial data volumes to achieve optimal performance^58^. To overcome this fundamental limitation, transfer learning emerges as a pivotal strategy. This approach enables us to leverage the rich, hierarchical feature representations learned by state-of-the-art CNN models pre-trained on large-scale datasets (e.g., ImageNet)^59^. Rather than learning from random initialization, we adapt these powerful feature extractors to our specific task of lung nodule classification. This paradigm leverages generalized visual knowledge (e.g., edge detection, texture patterns, shape recognition) acquired from natural images, which proves highly transferable to medical imaging domains.

In this study, we meticulously evaluated a diverse suite of modern architectures renowned for their efficacy in image classification. Through rigorous empirical analysis on our target dataset, we identified the six top-performing models to serve as the foundational feature extractors for our ensemble framework. The selected models are: EfficientNetB6, Inception v3, ResNet50, DenseNet201, DenseNet121, and MobileNetV2. This selection ensures a blend of architectural diversity covering residual connections, dense connectivity, compound scaling, and inverted residuals, which is crucial for capturing a comprehensive spectrum of discriminative features from lung CT scans. By initializing our framework with these pre-trained weights and subsequently optimizing them on the lung cancer dataset, we effectively harness the power of transfer learning.

#### EfficientNetB6-based model implementation

EfficientNet-B6 is recognized for delivering strong classification accuracy while maintaining computational efficiency, owing to its innovative compound scaling technique. This strategy uniformly scales the network’s depth, width, and input resolution, resulting in balanced performance improvements without unnecessary computational overhead. At the heart of the architecture is the Mobile Inverted Bottleneck Convolution (MBConv) block, which integrates SE mechanisms to adaptively recalibrate channel-wise feature responses. This feature refinement enables the model to better emphasize relevant patterns and suppress less critical information, enhancing its effectiveness in detecting nuanced features within cervical cytology images^60^.

EfficientNet’s robustness persists even when operating on lower-resolution images, making it a strong candidate for applications involving variable image quality. The architecture’s scalability is guided by a set of compound coefficients: a global scaling factor $\phi$, and constants $\alpha$, $\beta$, and $\gamma$, which define how resolution, width, and depth are scaled from the baseline configuration $r_0$, $w_0$, and $d_0$, respectively. These relationships are mathematically described in Eqs. (8), (9), and (10), ensuring a systematic approach to model scaling.

8 $$\begin{aligned} \text {Resolution:} \quad&r = \alpha ^{\phi } \cdot r_0 \end{aligned}$$

9 $$\begin{aligned} \text {Width:} \quad&w = \beta ^{\phi } \cdot w_0 \end{aligned}$$

10 $$\begin{aligned} \text {Depth:} \quad&d = \gamma ^{\phi } \cdot d_0 \end{aligned}$$

#### Inception v3 based model implementation

The Inception V3 model, developed by Google, represents an advanced version of the original Inception network. It was designed to increase classification accuracy while maintaining computational efficiency. The core idea behind the Inception architecture is to approximate an optimal sparse structure by transforming it into a dense one, where similar non-uniform sparse elements are clustered together. This approach enables the formation of deeper and wider networks without significantly increasing computational costs, as described in^61^. The architectural design of Inception V3 is based on several guiding principles:

1. Locality of information: Many signals in the input space tend to be spatially correlated. This allows for the use of smaller convolution filters to capture meaningful patterns. By applying dimension reduction (e.g., using $1 \times 1$ convolutions) before expensive operations, computational efficiency can be improved without significant information loss.
2. Balanced network expansion: To effectively utilize available computational resources, both the depth (number of layers) and width (number of filters) of the network should be increased in tandem. This enables the model to learn richer and more diverse feature representations.
3. Gradual dimensional reduction: Aggressive reduction in feature map dimensions at early stages of the network is discouraged, as it may lead to the loss of critical information. Instead, gradual downsampling is preferred to preserve spatial details during the early learning phase.
4. Wide layer efficiency: Wider layers tend to learn faster and are especially beneficial at deeper levels of the network, where abstract feature representations are formed.

#### ResNet50 based model implementation

ResNet50^62^, a popular and deeper variant of ResNet, comprises 50 layers and was initially trained on the ImageNet dataset, which includes over a million labeled images. Its architecture involves batch normalization layers, non-linearities, and a combination of identity and convolutional residual blocks. ResNet50 is frequently paired with other architectures, such as Highway Networks, which learn skip connections through additional weight matrices.

The network begins with a conv1 layer consisting of 64 filters using a $7\times 7$ kernel, followed by a max-pooling operation with a stride of 2. The subsequent layers conv2 through conv5 are composed of residual blocks. Each block includes three convolutional layers: two $1\times 1$ convolutions (first for dimensionality reduction, the second for restoration) and a $3\times 3$ convolution sandwiched between them, as described in Eq. 11. This structure is iteratively applied within each residual block, with the final convolutional stage feeding into an average pooling layer and a fully connected layer. Classification is performed using a Softmax activation function.

11 $$\begin{aligned} \begin{bmatrix} 1 \times 1 & 64 \\ 3 \times 3 & 64 \\ 1 \times 1 & 256 \end{bmatrix} \end{aligned}$$

#### DenseNet-121 based model implementation

The DenseNet-121 architecture^63^ is a key advancement in deep learning for computer vision, designed to enhance feature reuse and mitigate the vanishing gradient problem in very deep networks. Its hallmark is the dense connectivity pattern, where each layer receives feature maps from all preceding layers and passes its outputs to all subsequent ones. This design provides implicit deep supervision, strengthening feature propagation. The network is structured into multiple dense blocks, where each layer applies batch normalization, a Rectified Linear Unit (ReLU), and a $3 \times 3$ convolution, with outputs concatenated to preserve low-level details while building high-level representations. Between dense blocks, transition layers consisting of a $1 \times 1$ convolution for feature compression followed by $2 \times 2$ average pooling regulate spatial dimensions and computational cost. Overall, DenseNet-121 achieves strong performance while maintaining parameter efficiency through its compact yet expressive design.

#### DenseNet-201 based model implementation

Building upon the foundational DenseNet architecture^63^, DenseNet-201 represents a deeper and more powerful variant designed to further enhance feature representation and gradient flow in very deep convolutional networks. While it shares the core principles of dense connectivity and feature reuse with DenseNet-121, its increased depth makes it particularly adept at capturing complex, hierarchical patterns in medical images. The architecture is structured around four dense blocks interconnected by transition layers. The key differentiator from DenseNet-121 is its greater depth, achieved by incorporating more layers within these dense blocks. This enables the network to learn a more comprehensive hierarchy of features, ranging from simple edges and textures in the early layers to complex morphological structures in the deeper layers.

The hallmark dense connectivity remains central to its design: each layer within a block receives concatenated feature maps from all preceding layers and passes its own outputs to all subsequent layers. This design ensures:

- Maximized Feature Reuse: Low-level features, such as tissue textures or nodule edges, are preserved and utilized throughout the network, improving representational efficiency.
- Alleviated Vanishing Gradient: The direct connections provide shortened paths for gradient flow during backpropagation, ensuring stable training even at this significant depth.
- Implicit Deep Supervision: The feature reuse acts as a form of regularization, reducing the risk of overfitting on limited medical datasets.

#### MobileNetV2 based model implementation

To incorporate a highly efficient architecture into our ensemble, we selected MobileNetV2^64^. This model was specifically designed for mobile and embedded vision applications, offering an excellent trade-off between computational efficiency and accuracy. Its core innovation is the inverted residual with a linear bottleneck structure. Unlike traditional residual blocks that first compress and then expand feature maps, MobileNetV2’s inverted residual block first expands the channel dimension using a lightweight $1 \times 1$ convolution. This expansion is followed by a depthwise convolution that filters the expanded features, and a final $1 \times 1$ convolution that projects the features back to a lower-dimensional representation. This design significantly reduces computational cost and model size while preserving critical information. A key feature of this architecture is the use of linear bottlenecks. Instead of applying a non-linear activation function (such as ReLU) after the final projection layer, a linear activation function is used. This prevents non-linearities from destroying important information in the low-dimensional space, leading to more stable and representative features.

### Proposed enhanced animated oat optimization algorithm with genetic operators (EAOO-GA)

While the standard Animated Oat Optimization (AOO) algorithm demonstrates innovative inspiration from natural phenomena, our empirical analysis reveals certain limitations that can hinder its performance on complex optimization problems, such as those encountered in medical image analysis. Primarily, the algorithm can occasionally exhibit a tendency towards premature convergence, where the population loses diversity too rapidly and becomes trapped in local optima. This is often attributed to its strong exploitation bias in the later stages, without a sufficient mechanism to reintroduce lost diversity or to escape suboptimal regions once discovered. To mitigate these limitations and enhance the algorithm’s robustness and global search capability, we propose an Enhanced Animated Oat Optimization (EAOO-GA) algorithm. The core innovation lies in the strategic integration of genetic operators, including crossover and mutation, into the standard AOO workflow. This hybrid approach leverages the strength of AOO’s bio-inspired exploration and exploitation while bolstering its population diversity through well-established evolutionary mechanisms.

#### Standard AOO algorithm

The AOO is a new metaheuristic algorithm that emulates three specific behaviors observed in the natural conduct of animated oats within their environment^65^.

1. Initial seed displacement through environmental forces, including wind, water currents, and animal-mediated transport.
2. Moisture-induced morphological changes in the seed’s awn structure generate mechanical forces that produce rolling motion across terrestrial surfaces.
3. Kinetic energy accumulation during locomotion phases, with obstacle interactions triggering mechanical energy release mechanisms for enhanced dispersal range.

***Initialization*** AOO initializes its population by generating a set of random solutions, as formalized in Eq. 12

12 $$\begin{aligned} X=\begin{bmatrix} X_{1,1} & ... & X_{1,j} & ... & X_{1,Dim-1}& X_{1,Dim}\\ X_{2,1} & ... & X_{2,j} & ... & X_{2,Dim-1}& X_{2,Dim}\\ ..& ..& .. & ..& ..& ..\\ ..& ..& X_{i,j} & ..& ..& ..\\ ..& ..& .. & ..& ..& ..\\ X_{N-1,1} & ... & X_{N-1,j} & ... & X_{N-1,Dim-1}& X_{N-1,Dim} \\ X_{N,1} & ... & X_{N,j} & ... & X_{N,Dim-1}& X_{N,Dim} \\ \end{bmatrix} \end{aligned}$$

The position of each individual in the $i^{\text {th}}$ subgroup is denoted by $\textbf{x}i$. The subpopulation size and the problem’s dimension are given by *N* and *Dim*, respectively. Each element *x* *i*, *j* of the position matrix $\textbf{X}$ is computed using Eq. 13:

13 $$\begin{aligned} x_{i,j} = r * ( UB_j - LB_j ) + LB_j, i = 1, 2, \dots , N, j = 1, 2, \dots ,Dim \end{aligned}$$

where *r* is a random number between 0 and 1.

Parameter Calculation The dynamic behavior of the animated oat during dispersal is characterized by three key biomechanical parameters: seed mass, eccentric rolling coefficient, and primary awn length. These parameters are algorithmically computed as follows:

14 $$\begin{aligned} \left\{ \begin{matrix}m = 0.5 * \frac{r}{Dim}\\ \\ L = N * \frac{r}{Dim} \\ \\ e = 0.5 *\frac{r}{Dim} \\ \\ c = 1 -\left( \frac{t}{T} \right) ^3 \\ \end{matrix} \right\} \end{aligned}$$

where *m* is the seed mass, *N* is the population size, *L* is the primary awn length, *e* is the eccentric rotation coefficient, *t* is the current iteration, and *c* is a dynamic adjustment factor.

Exploration Phase Following abscission, the dispersal of animated oat seeds is primarily driven by environmental vectors such as wind, water, or fauna. The stochastic nature of this process facilitates extensive exploration of the search space. The corresponding position update is defined as:

15 $$\begin{aligned} W= & \frac{c}{\pi } * (2 * r_{Dim} - 1) \otimes UB \end{aligned}$$

16 $$\begin{aligned} X_{t+1}(i)= & {\left\{ \begin{array}{ll} \frac{1}{N} \sum _{j=1}^{N} X_t(j) + W, & \text {if } \mod (i, N/10) = 0, \\ X_{\text {best}} + W, & \text {if } \mod (i, N/10) = 1, \\ X_t(i) + W, & \text {otherwise}. \end{array}\right. } \end{aligned}$$

where $X_{t+1}(i)$ and $X_{\text {best}}$ denote the positions of the $i^{\text {th}}$ individual and the best individual in the population at iteration $t + 1$, respectively.

Exploitation Phase The remaining seeds are partitioned into two subsets based on the presence of obstacles during dispersal, assuming both outcomes are equiprobable. In the absence of obstructions, seeds undergo hygroscopic rolling driven by moisture-induced stress gradients. This motion is modeled using curvature-induced snap buckling, inspired by the work of Lindtner et al.^66^, which demonstrated that anisotropic swelling is governed by cellulose microfiber orientation. The rolling dynamics are mathematically represented through torque equations and eccentric rotation:

17 $$\begin{aligned} A= & UB - \left| \frac{UB * t * sin(2 * \pi * r)}{T}\right| \end{aligned}$$

18 $$\begin{aligned} R= & ( m * e + L^2) *\frac{r_{Dim}*(- A,A)}{Dim} \end{aligned}$$

19 $$\begin{aligned} Levy(dim)= & 0.01 * \frac{\mu * \sigma }{\left| \nu ^\frac{1}{\beta } \right| } \end{aligned}$$

20 $$\begin{aligned} \sigma= & \left( \frac{\Gamma (1+\beta )* sin(\frac{\pi *\beta }{2})}{\Gamma (\frac{1+\beta }{2})*\pi *2^{(\frac{\beta -1}{2})}} \right) ^\frac{1}{\rho } \end{aligned}$$

21 $$\begin{aligned} X_t(i)= & X_{best}+ R + c * Levy(Dim) \otimes X_{best} \end{aligned}$$

where $r_{Dim}(-A, A)$ is a random matrix of size *Dim* with entries uniformly distributed in $(-A, A)$. The mean value $\mu$ in the Lévy flight is a random number between 0 and 1, typically used to adjust the step size. This stochasticity helps regulate the direction and distance of movement during flight. The scale parameter $\sigma$ controls the width of the step length distribution, defining the range of possible step sizes. The current velocity vector $\nu$ represents the motion state of the particle or individual. The stability parameter $\beta \in (0,2]$ governs the shape of the distribution, influencing the diversity and randomness of step lengths. The gamma function $\Gamma$ generalizes the factorial function to continuous values. For Lévy flights, $\beta$ is typically set to 1.5. The position update is defined as follows:

22 $$\begin{aligned} & B = UB - \left| \frac{UB * t * cos(2 * \pi * r)}{T}\right| \end{aligned}$$

23 $$\begin{aligned} & \left\{ \begin{matrix}k = 0.5 + 0.5 * r\\ \\ x = 3 * \frac{r}{Dim} \\ \\ \theta = \pi * r \\ \\ \alpha = \frac{1}{\pi }* e^{\frac{r^{\prime }}{T}} \\ \end{matrix} \right\} \end{aligned}$$

24 $$\begin{aligned} & J=\frac{2*k*x^2*sin(2\theta )}{mg} * \frac{r_{Dim}*(-B, B)}{Dim} * (1-\alpha ) \end{aligned}$$

25 $$\begin{aligned} & X_t(i) = X_{best}+ J + c * Levy(Dim) \otimes X_{best} \end{aligned}$$

where $\theta$ denotes the launch angle relative to the ground, $\alpha$ is the air drag coefficient governing aerial motion, $r' \in [0, T]$ is a uniformly sampled random value, *k* quantifies the elasticity of the primary awn, and *x* represents the change in awn length due to elastic energy storage prior to release.

#### EAOO-GA algorithm

To address the limitations of the standard AOO, we introduce two genetic operators applied after the core AOO update procedures. These operators serve as a diversity-preserving mechanism, enabling the algorithm to avoid premature convergence and explore the search space more effectively.

Crossover Operator We employ a differential crossover mechanism to facilitate the exchange of information between individuals. For each target individual $X_i$ in the population, a trial vector $U_i$ is generated by combining components from the target vector and a mutant vector $V_i$ created from three distinct randomly selected individuals:

26 $$\begin{aligned} V_i= & X_{r1} + F \cdot (X_{r2} - X_{r3}) \end{aligned}$$

27 $$\begin{aligned} U_{i,j}= & {\left\{ \begin{array}{ll} V_{i,j} & \text {if } rand(0,1) \le CR \text { or } j = j_{rand} \\ X_{i,j} & \text {otherwise} \end{array}\right. } \end{aligned}$$

where $X_{r1}$, $X_{r2}$, $X_{r3}$ are distinct random individuals, *F* is a scaling factor, *CR* is the crossover rate, and $j_{rand}$ ensures at least one parameter is inherited from the mutant vector.

Mutation Operator A non-uniform mutation strategy is applied to introduce random perturbations, with decreasing magnitude over iterations to transition from exploration to exploitation:

28 $$\begin{aligned} X_{i,j}^{new} = {\left\{ \begin{array}{ll} X_{i,j} + \Delta (t, UB_j - X_{i,j}) & \text {if } rand() < 0.5 \\ X_{i,j} - \Delta (t, X_{i,j} - LB_j) & \text {otherwise} \end{array}\right. } \end{aligned}$$

where $\Delta (t, y)$ calculates the mutation step size that decreases over time:

29 $$\begin{aligned} \Delta (t, y) = y \cdot \left( 1 - r^{(1 - t/T)^b}\right) \end{aligned}$$

Here, *r* is a random number in [0, 1], *T* is the maximum iterations, and *b* determines the non-uniformity degree.

Selection and Integration The genetic operators are applied after AOO’s exploitation phase. A greedy selection mechanism determines whether newly generated solutions replace existing ones:

30 $$\begin{aligned} X_i^{t+1} = {\left\{ \begin{array}{ll} U_i & \text {if } f(U_i) \le f(X_i) \\ X_i & \text {otherwise} \end{array}\right. } \end{aligned}$$

This hybrid approach maintains AOO’s bio-inspired search capabilities while enhancing its global optimization performance through evolutionary mechanisms. The pseudocode of EAOO-GA is provided in Algorithm 1.

**Algorithm 1:**
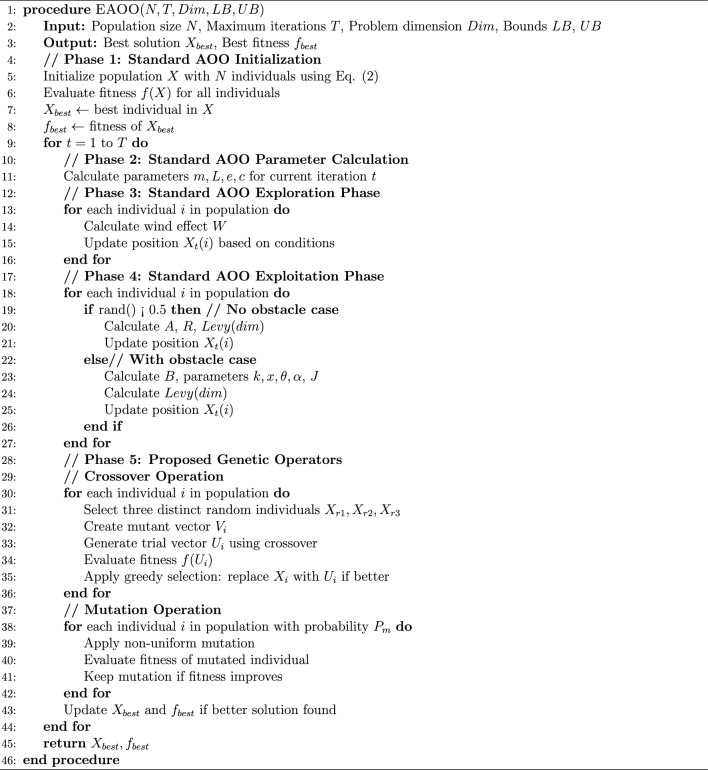
Pseudocode of Enhanced Animated Oat Optimization (EAOO-GA)

### The feature fusion framework

In this study, we propose an optimized ensemble framework that extends beyond traditional model aggregation. Rather than merely combining outputs from individual models, we employ feature fusion architectures that integrate complementary representations from multiple deep learning backbones to enhance accuracy. Three tailored fusion architectures: Fusion 1, Fusion 2, and Fusion 3 were developed, each designed to exploit the distinctive strengths of different pre-trained models.

The selection of models for these architectures followed a systematic process designed to maximize both diversity and efficiency. To prevent redundancy and capture a broader range of feature representations, models from the same architectural family were not combined. Instead, our fusion strategy paired networks of differing capacities and inductive biases, specifically, coupling a high-capacity model with a more lightweight counterpart to balance diagnostic accuracy with computational cost. The resulting three fusion pairs are as follows:

- **Fusion 1:** DenseNet201 + EfficientNetB6
- **Fusion 2:** Inception v3 + MobileNetV2
- **Fusion 3:** DenseNet121 + ResNet50

**Fig. 5:**
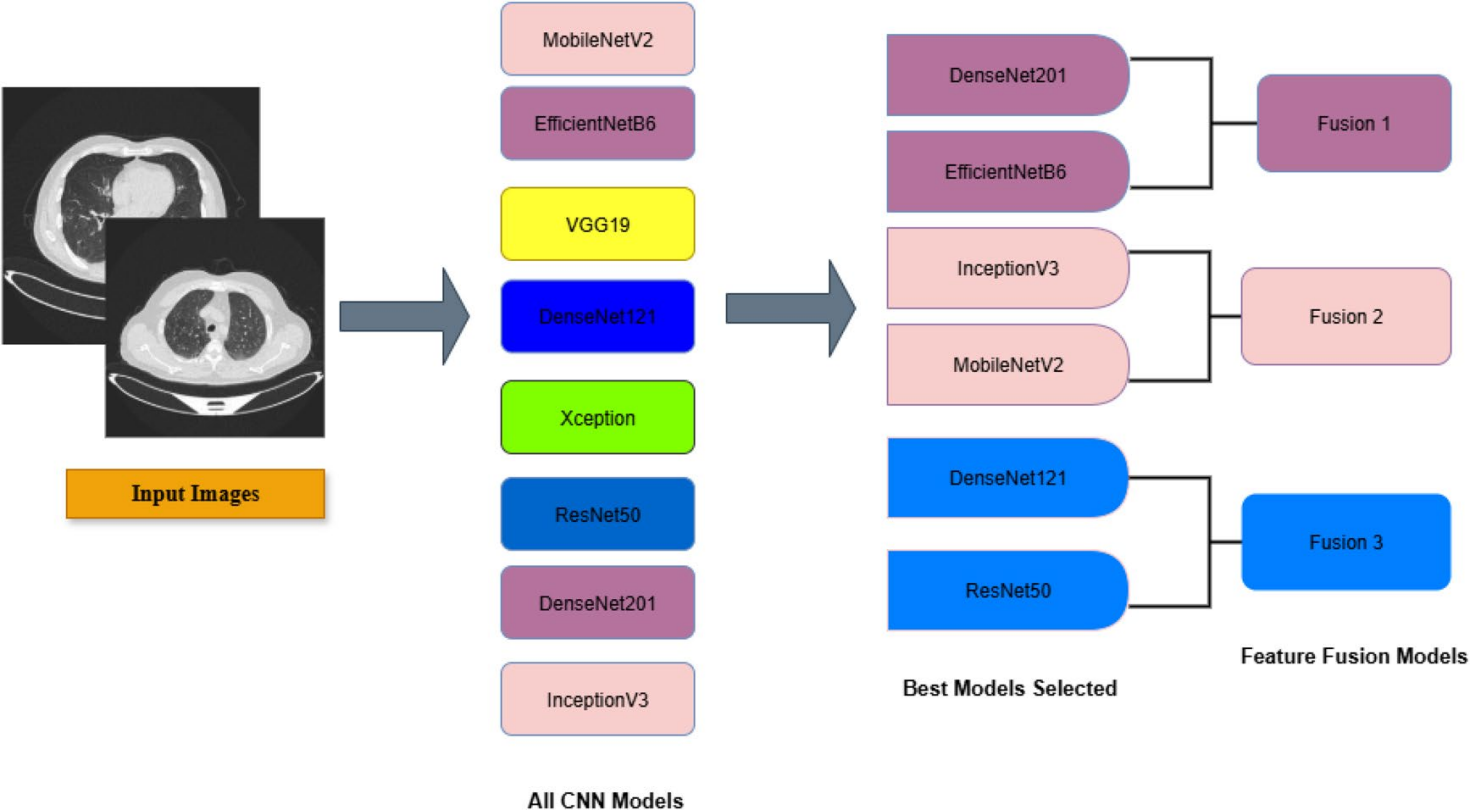
Selection pipeline of candidate models for feature fusion.

The feature extraction process begins by propagating input images through the pre-trained models within each fusion pair. The resulting feature maps are then concatenated, integrating the distinct hierarchical patterns learned by each network. This systematic selection process yielded an initial pool of eight pre-trained models. Through rigorous evaluation, the six top-performing models were identified and strategically paired to form the three distinct fusion architectures, as illustrated in the model selection pipeline (Fig. 5). To strengthen the discriminative capacity of the fused representations, we incorporate an SE block. This mechanism adaptively recalibrates channel-wise responses, enabling the network to highlight informative features, such as texture and morphological patterns associated with malignancy, while suppressing irrelevant ones. Such dynamic feature emphasis is especially vital in lung cancer detection, where subtle yet critical visual cues must be accurately identified.

**Fig. 6:**
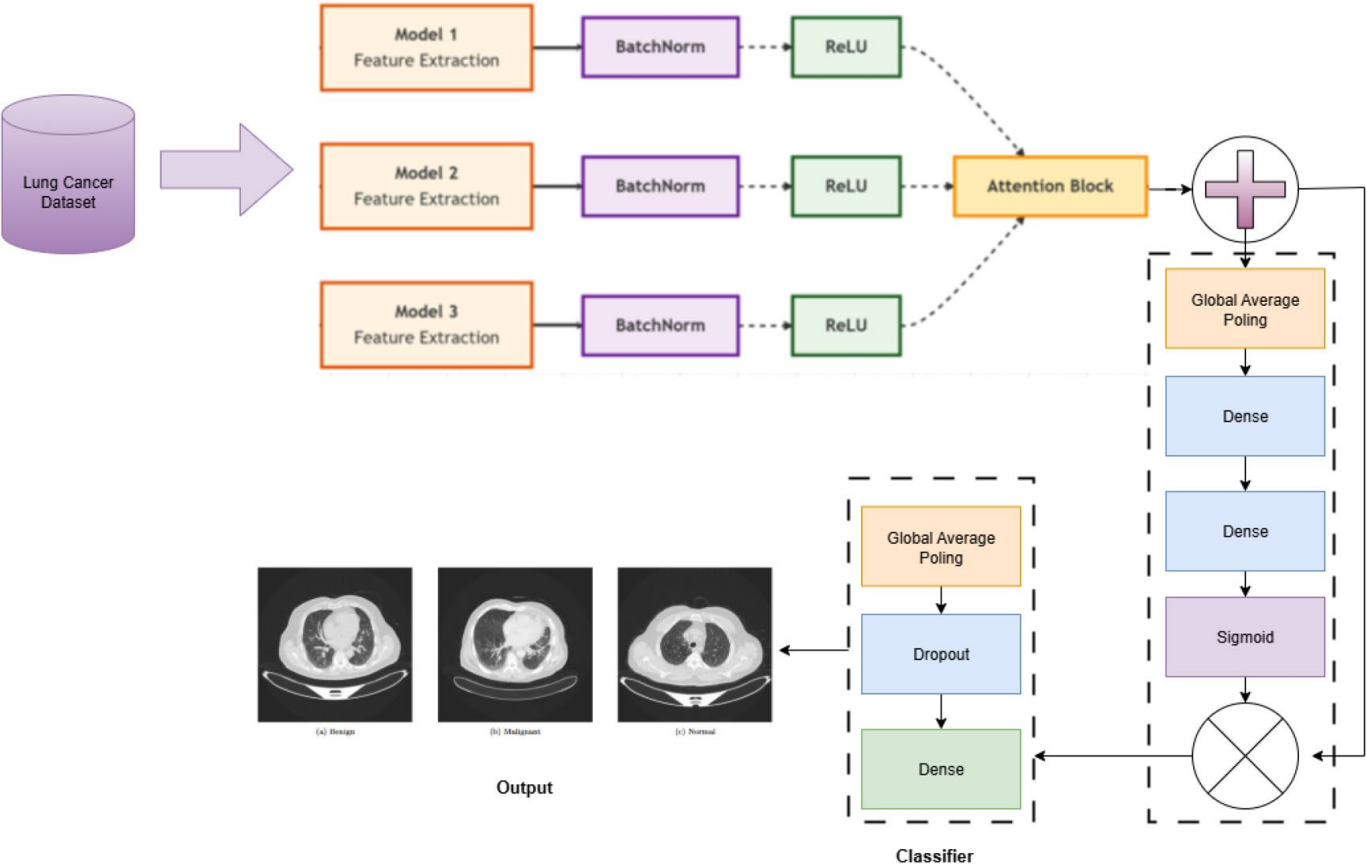
Block diagram of the feature fusion architectures.

The recalibrated features from the SE block are subsequently subjected to Global Average Pooling, which reduces dimensionality while preserving salient feature information and enhancing robustness to spatial translations. A Dropout layer is strategically inserted following this operation to serve as a regularization mechanism, effectively mitigating overfitting by preventing complex co-adaptations of features during training. The network culminates in a softmax activation layer, which generates the final probability distribution across the target diagnostic classes for each fusion pathway. The block diagram of the feature fusion architectures is shown in Fig. 6. This feature fusion methodology is fundamentally motivated by the need to compensate for the performance variability and inherent limitations of individual models. By harnessing complementary representations from multiple architectural families, our ensemble framework achieves synergistic improvements in predictive accuracy, generalization capability, and operational robustness for pulmonary nodule classification. The integrated approach provides three distinct advantages: (1) superior classification performance through multi-perspective feature integration, (2) expanded representational capacity for capturing heterogeneous pathological patterns, and (3) inherent interpretability facilitated by the SE block’s channel-wise attention mechanism, which collectively contribute to more reliable and clinically actionable diagnostic support.

### Proposed SE-FusionEAOO ensemble framework for lung cancer detection

In this section, we present a novel hybrid optimized ensemble framework tailored for accurate and reliable lung cancer detection from CT scans. Ensemble learning is particularly advantageous in medical diagnostics, as it enhances predictive accuracy and robustness by combining the strengths of multiple diverse models^67^. Such reliability is critical in lung cancer detection, where diagnostic errors may have serious consequences. The proposed framework is designed to achieve superior classification performance through two stages: (i) constructing three SE-enhanced fusion models as strong base learners, and (ii) aggregating their outputs using an optimized weighting strategy. The distinguishing feature of our approach lies in employing the Enhanced Animated Oat Optimization algorithm (EAOO-GA), described in Section 3.4, to fine-tune the ensemble weights. This metaheuristic efficiently explores the global search space to identify the optimal weight configuration, ensuring that the most informative models contribute more prominently to the final decision. As a result, the system achieves higher reliability and diagnostic precision. The overall architecture of the proposed ensemble is depicted in Fig. 1.

#### Ensemble construction and optimization process

The process for building and optimizing our ensemble is methodically outlined below and summarized in Algorithm 2.

**Step 1: Base Model Training and Feature Fusion** Three separate fusion models (Fusion 1, Fusion 2, Fusion 3) are constructed by pairing different pre-trained architectures (DenseNet201+EfficientNetB6, Inception v3+MobileNetV2, DenseNet121+ResNet50). Each pair is integrated using concatenation and enhanced with SE blocks for adaptive feature recalibration, as described in Section 3.2. These models are trained on the lung cancer dataset to serve as expert feature extractors and classifiers.

**Step 2: Prediction Generation** Each of the three trained fusion models is used to generate prediction vectors (e.g., class probabilities for ’Benign’, ’Malignant’, ’Normal’) on the validation or test set. Let $P_1, P_2, P_3$ represent the prediction matrices from Fusion 1, Fusion 2, and Fusion 3, respectively.

**Step 3: Fitness Function Definition** The core objective of the EAOO algorithm is to find the optimal weight vector $\textbf{w} = [w_1, w_2, w_3]$ that maximizes the accuracy of the weighted ensemble prediction. The fitness function is defined as:

31 $$\begin{aligned} \text {Fitness}(\textbf{w}) = \text {Accuracy}\left( \text {argmax}\left( \sum _{i=1}^{3} w_i \cdot P_i \right) , Y_{\text {true}} \right) \end{aligned}$$

where $Y_{\text {true}}$ are the true labels, and $\sum w_i = 1$, $w_i \ge 0$.

**Step 4: Weight Optimization via EAOO** The proposed EAOO algorithm is deployed to solve this optimization problem. The EAOO population consists of candidate weight vectors. The algorithm evolves these candidates over generations through its operations (exploration, exploitation, crossover, mutation) to maximize the fitness function, ultimately converging on the optimal set of weights $\mathbf {w^*}$.

**Step 5: Final Ensemble Prediction** Once optimized, the final prediction for a new input image is made by a weighted average of the base model predictions using the optimal weights $\mathbf {w^*}$:

32 $$\begin{aligned} P_{\text {final}} = w_1^* \cdot P_1 + w_2^* \cdot P_2 + w_3^* \cdot P_3 \end{aligned}$$

The class with the highest probability in $P_{\text {final}}$ is selected as the final diagnosis.

Our EAOO-optimized ensemble framework provides a dynamic and highly accurate solution for lung nodule classification. By synergistically combining the representational power of multiple deep fusion models with the global optimization capability of EAOO-GA, we achieve a system that is not only more accurate than its individual components but also inherently robust and reliable. This approach underscores the significant potential of integrating advanced meta-heuristic optimization with deep ensemble learning to tackle critical challenges in medical image analysis.

**Algorithm 2:**
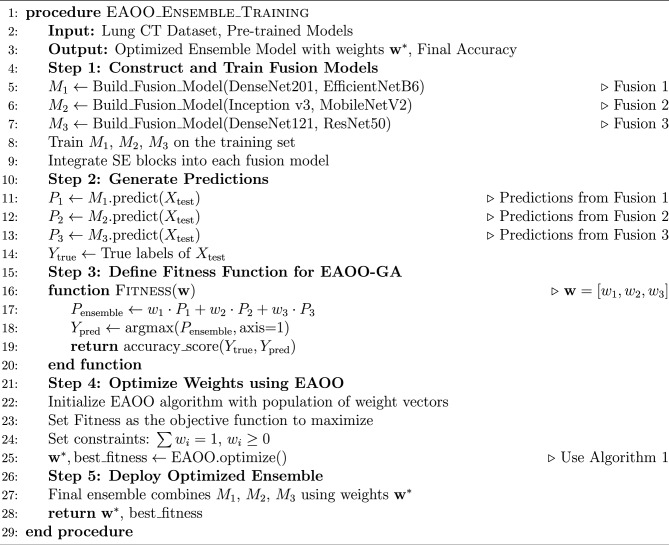
Training and Optimization Process of the Proposed Ensemble

#### Integration of explainable AI (XAI)

The term “explainable artificial intelligence” (XAI) refers to a range of methods designed to elucidate and illustrate how complex AI models make decisions. In this paper, we employed the Gradient-weighted Class Activation Mapping (Grad-CAM) technique to enhance the interpretability of our proposed cervical cancer classification framework. Grad-CAM generates class-specific heatmaps that visually highlight the most influential regions in input images, enabling clinicians to verify the model’s focus and improve transparency in the diagnostic process^68^.

***Grad-CAM***: This technique generates a local visual explanation by utilizing the target class gradients flowing to the last convolutional layer, creating an approximate localization map at the end of the prediction stage, as described in Eq. (33).

33 $$\begin{aligned} \mathcal {M}_c^{\text {CAM}} = \textrm{ReLU} \left( \sum _{m} \gamma _m^c \cdot \mathcal {F}^m \right) \end{aligned}$$

In this expression, $\mathcal {M}_c^{\text {CAM}}$ denotes the class activation map associated with category $c$. The operator $\textrm{ReLU}(\cdot )$ ensures that only positively contributing features are retained by zeroing out negative values. $\mathcal {F}^m$ refers to the activation output from the $m$-th channel of the final convolutional layer, while $\gamma _m^c$ signifies the importance score of this channel for the target class $c$. This score is determined by computing the spatial average of the gradient of the class score with respect to the feature map:

34 $$\begin{aligned} \gamma _m^c = \frac{1}{H \cdot W} \sum _{p=1}^{H} \sum _{q=1}^{W} \frac{\partial \hat{y}_c}{\partial \mathcal {F}_{p,q}^m} \end{aligned}$$

Here, $\hat{y}_c$ is the network’s raw output (logit) for class $c$, and $\mathcal {F}_{p,q}^m$ represents the activation value at position $(p, q)$ in the $m$-th feature map. The denominator $H \cdot W$ corresponds to the total number of spatial locations in the feature map and acts as a normalization factor. The resulting heatmap $\mathcal {M}_c^{\text {CAM}}$ visually emphasizes image regions most influential in the network’s decision-making process for class $c$.

### Computational complexity

The proposed SE-FusionEAOO Ensemble framework involves several components, each contributing to the overall time complexity. Feature extraction from the pre-trained CNN pairs dominates during training and inference, with a per-image complexity of $O(N \cdot C \cdot H \cdot W)$ for convolutional operations, where N is the batch size, *C* the number of channels, and *H*/*W* the spatial dimensions (typically $224 \times 224$). The SE blocks add a lightweight overhead of $O(C^2 / r)$ per block, with *r* the reduction ratio (default 16), ensuring minimal impact. SMOTE for handling class imbalance has a preprocessing time complexity of $O(D \cdot M \log M)$, where *D* is the feature dimensionality and *M* the number of minority samples. The EAOO-GA metaheuristic for weight optimization has a time complexity of $O(P \cdot G \cdot (E + F))$, where *P* is the population size, *G* the number of generations, *E* the time for ensemble prediction on the dataset (proportional to the sum of base model complexities), and *F* the fitness computation. Overall, the framework’s time complexity is dominated by the CNN forward passes and EAOO-GA iterations; however, it remains practical for medical imaging tasks due to the transfer learning and parallel computation capabilities.

### Mathematical formulation of the proposed framework

Let $\textbf{I}$ denote an input CT image and $\Phi _k(\textbf{I}) \in \mathbb {R}^{d_k}$ represent the deep feature vector extracted from the $k^{\text {th}}$ pretrained backbone ($k=1,2,3,4,5,6$). For each fusion branch, feature maps from two complementary networks are concatenated and refined through an SE block to obtain channel-wise recalibrated features:

35 $$\begin{aligned} \textbf{F}_k = \text {SE}([\Phi _{a_k}(\textbf{I}) \, \Vert \, \Phi _{b_k}(\textbf{I})]), \end{aligned}$$

where $[\cdot \Vert \cdot ]$ denotes concatenation and $\text {SE}(\cdot )$ represents the excitation function defined by:

36 $$\begin{aligned} \text {SE}(\textbf{z}) = \sigma (W_2 \, \delta (W_1 \, \text {GAP}(\textbf{z}))), \end{aligned}$$

with $\text {GAP}(\cdot )$ being global average pooling, $\delta (\cdot )$ the ReLU activation, $\sigma (\cdot )$ the sigmoid gating, and $W_1$, $W_2$ learnable parameters. The ensemble output combines the softmax probabilities of *M* fusion experts, each producing a class-score vector $\textbf{p}_k \in \mathbb {R}^{C}$:

37 $$\begin{aligned} \textbf{P}_{ens} = \sum _{k=1}^{M} w_k \, \textbf{p}_k, \quad \text {s.t. } \sum _{k=1}^{M} w_k = 1, \; w_k \ge 0. \end{aligned}$$

To determine the optimal contribution of each fusion model, the EAOO-GA algorithm minimizes the cross-entropy-based objective function:

38 $$\begin{aligned} f(\textbf{w}) = -\frac{1}{N}\sum _{i=1}^{N} \sum _{c=1}^{C} y_{i,c}\log (P_{ens}^{(c)}(\textbf{I}_i;\textbf{w})), \end{aligned}$$

where $y_{i,c}$ is the ground-truth label and $P_{ens}^{(c)}$ is the predicted probability for class *c*. Within EAOO-GA, the population of candidate weight vectors $\textbf{w}_i^t$ evolves iteratively via the animated oat dynamic operators (Eqs. 16–25) and the incorporated genetic crossover and mutation (Eqs. 27–29).

The best solution $\textbf{w}^*$ at convergence satisfies:

39 $$\begin{aligned} \textbf{w}^* = \arg \min _{\textbf{w}} f(\textbf{w}), \end{aligned}$$

yielding the final optimized ensemble decision:

40 $$\begin{aligned} \hat{y} = \arg \max _{c} P_{ens}^{(c)}(\textbf{I};\textbf{w}^*). \end{aligned}$$

This mathematical formulation clarifies how the proposed framework unifies multi-model feature fusion, attention-based recalibration, and metaheuristic weight optimization into a coherent and analytically defined learning pipeline.

## Results and discussion

This section presents a comprehensive evaluation of the proposed SE-FusionEAOO Ensemble framework. We begin by detailing the experimental setup and hyperparameter configurations. The model’s performance is then rigorously assessed on the IQ-OTH/NCCD lung cancer dataset, demonstrating its diagnostic capabilities. To enhance interpretability, we employ Grad-CAM visualizations to elucidate the model’s decision-making process by highlighting critical regions in CT scans. A comparative analysis against both traditional optimization algorithms and state-of-the-art methods further establishes the superiority of the proposed approach. Collectively, these analyses provide compelling evidence for the efficacy and advancement of the SE-FusionEAOO Ensemble model in lung cancer detection.

### Implementation environment

All experiments were performed using Google Colab Notebook, an open-source cloud-based development platform that provides a suitable environment for executing ML workflows. The model was implemented using the Keras high-level API, with TensorFlow serving as the backend framework. The hardware configuration included a 10^th^ Generation Intel^®^ Core™ i9 processor, 32  GB of RAM, and a 64-bit Windows 10 operating system for initial code development and testing. Python version 3.6.9 was used as the primary programming language. In addition to Keras and TensorFlow, essential libraries such as NumPy, OpenCV, scikit-learn, and Matplotlib were employed for data preprocessing. To ensure reproducibility and consistent training across all models, the following hyperparameters were uniformly applied to the individual pre-trained models, SE-enhanced fusion architectures, and the final ensemble fine-tuning:

- **Optimizer**: Adam (with default $\beta _1=0.9$, $\beta _2=0.999$)
- **Learning Rate**: 1e-4 (chosen to balance convergence speed and stability in transfer learning on medical images, following common practice)
- **Batch Size**: 32 (suitable for GPU memory constraints while providing stable gradients)
- **Epochs**: Maximum 100, with Early Stopping (patience=10, monitor=’val_loss’) to prevent overfitting
- **Loss Function**: Categorical Cross-Entropy
- **Data Split**: 70% training, 15% validation, 15% testing (stratified to preserve class distribution)
- **Random Seed**: Fixed at 42 for TensorFlow, NumPy, and Python random operations to ensure reproducible results
- **Cross-Validation**: 5-fold stratified cross-validation used during model selection to robustly rank the eight candidate models

### Evaluation metrics

The performance of the proposed framework is evaluated using standard classification metrics, including accuracy, precision, recall, and F1-score. Together, these metrics provide a comprehensive assessment of the model’s classification effectiveness. Table 4 summarizes the metrics used in this study.

**Table 4:** Overview of evaluation metrics used to assess classification performance in deep learning models.

Metric	Formula	Explanation
Accuracy	$\frac{TP + TN}{TP + TN + FP + FN}$	Ratio of all correct predictions (positive and negative) to the total number of samples.
Precision	$\frac{TP}{TP + FP}$	Percentage of true positives among all instances predicted as positive.
Recall	$\frac{TP}{TP + FN}$	Percentage of actual positive instances that were correctly predicted.
F1 Score	$\frac{2 \cdot \text {Precision} \cdot \text {Recall}}{\text {Precision} + \text {Recall}}$	Harmonic mean of precision and recall, used to balance the two metrics.

Where $TP$ indicates the number of true positives, $TN$ refers to true negatives, $FP$ denotes false positives, and $FN$ corresponds to false negatives. Additionally, $y_i$ represents the actual label (ground truth) for the $i^\text {th}$ instance, $\hat{y}_i$ is the corresponding predicted label generated by the model, and $n$ signifies the total number of data samples used in the evaluation process.

### Performance evaluation of basic models and feature fusion impact

To ensure the statistical robustness and reliability of our evaluation, all experiments were conducted using 5-fold stratified cross-validation. Reported values represent the mean ($\mu$) and standard deviation ($\sigma$) of each metric across the 5 independent folds. Tables 5 and 6 summarize the results before and after applying data augmentation and SMOTE.

Tables 5 and 6 present a comprehensive comparison of the classification performance for individual baseline networks, proposed feature fusion architectures, and the final EAOO-optimized ensemble model. All reported values represent the mean ± standard deviation over five independent cross-validation folds, ensuring a statistically reliable evaluation.

Before addressing class imbalance (Table 5), the single CNN backbones exhibited noticeable variation in performance. DenseNet201 achieved the highest mean accuracy of $92.5\%\!\pm \!0.6$ and F1-score of $92.8\%\!\pm \!0.7$, demonstrating strong representational capacity for lung nodule discrimination. Conversely, lighter or less expressive architectures such as VGG19 and Xception achieved considerably lower accuracies of $80.2\%\!\pm \!1.6$ and $83.1\%\!\pm \!1.2$, respectively, confirming the dependence of diagnostic performance on network depth and architectural suitability. All three proposed fusion models consistently outperformed individual baselines, indicating the advantage of complementary feature integration. Among them, Fusion 1 (DenseNet201 + EfficientNetB6) yielded the best pre-SMOTE performance with $94.2\%\!\pm \!0.4$ accuracy.

After applying data augmentation and SMOTE (Table 6), overall performance improved markedly across all methods, especially for minority classes (“Benign” and “Normal”). The weakest baseline models (VGG19 and Xception) increased their accuracies to $84.5\%\!\pm \!1.6$ and $87.3\%\!\pm \!1.2$, respectively, yet still lagged behind the hybrid models. The proposed fusion architectures achieved further gains, with Fusion 1 reaching $96.5\%\!\pm \!0.4$ accuracy and $96.4\%\!\pm \!0.5$ F1-score, confirming the effectiveness of combining diverse feature encoders.

Most notably, the proposed SE-FusionEAOO Ensemble attained a mean accuracy of $99.4\%\!\pm \!0.2$ and an F1-score of $99.3\%\!\pm \!0.2$, outperforming all competing models by a statistically significant margin. The extremely low standard deviation values reflect consistent and stable generalization across folds. These findings demonstrate that the EAOO-GA optimization effectively assigns adaptive weights to the constituent models, maximizing their complementary strengths while mitigating individual weaknesses. Consequently, the framework establishes a new state-of-the-art benchmark on the IQ-OTH/NCCD dataset for lung cancer classification.

**Table 5:** Performance evaluation on the IQ-OTH/NCCD dataset before applying data augmentation and SMOTE (mean ± SD over 5 folds).

Model	Accuracy (%)	Precision (%)	Recall (%)	F1-Score (%)
DenseNet201	92.5 ± 0.8	91.8 ± 0.9	93.2 ± 0.7	92.8 ± 0.8
EfficientNetB6	90.1 ± 1.0	89.5 ± 0.8	90.8 ± 1.2	90.1 ± 0.9
InceptionV3	89.8 ± 1.1	88.9 ± 1.0	90.5 ± 0.9	89.7 ± 1.0
ResNet50	87.3 ± 1.3	86.1 ± 1.1	88.0 ± 1.0	87.0 ± 1.2
DenseNet121	88.4 ± 1.0	87.2 ± 0.9	89.1 ± 1.1	88.1 ± 0.9
MobileNetV2	85.2 ± 1.5	83.8 ± 1.3	86.0 ± 1.2	84.9 ± 1.4
VGG19	80.2 ± 1.8	78.5 ± 1.6	81.0 ± 1.4	79.7 ± 1.5
Xception	83.1 ± 1.7	81.9 ± 1.5	84.0 ± 1.6	82.9 ± 1.6
Fusion 1 (DenseNet201 + EfficientNetB6)	94.2 ± 0.7	93.5 ± 0.6	94.9 ± 0.8	94.2 ± 0.7
Fusion 2 (InceptionV3 + MobileNetV2)	93.5 ± 0.8	92.7 ± 0.9	94.2 ± 0.7	93.4 ± 0.8
Fusion 3 (DenseNet121 + ResNet50)	92.8 ± 0.9	91.9 ± 0.7	93.5 ± 0.9	92.7 ± 0.8
**EAOO-Optimized Ensemble**	**96.2** ± **0.5**	**95.8** ± **0.4**	**96.5** ± **0.5**	**96.1** ± **0.4**

**Table 6:** Performance evaluation on the IQ-OTH/NCCD dataset **after** applying data augmentation and SMOTE (mean ± SD over 5 folds).

Model	Accuracy (%)	Precision (%)	Recall (%)	F1-Score (%)
DenseNet201	95.1 ± 0.6	94.3 ± 0.5	95.8 ± 0.6	95.0 ± 0.6
EfficientNetB6	93.2 ± 0.8	92.4 ± 0.7	93.9 ± 0.8	93.1 ± 0.7
InceptionV3	92.8 ± 0.9	91.9 ± 0.8	93.5 ± 0.9	92.7 ± 0.8
ResNet50	90.5 ± 1.0	89.4 ± 0.9	91.2 ± 1.0	90.3 ± 0.9
DenseNet121	92.1 ± 0.7	91.0 ± 0.6	92.8 ± 0.8	91.9 ± 0.7
MobileNetV2	89.3 ± 1.1	88.1 ± 0.9	90.0 ± 1.0	89.0 ± 1.0
VGG19	84.5 ± 1.6	83.1 ± 1.3	85.5 ± 1.4	84.3 ± 1.5
Xception	87.3 ± 1.2	86.0 ± 1.0	88.2 ± 1.2	87.1 ± 1.1
Fusion 1 (DenseNet201 + EfficientNetB6)	96.5 ± 0.4	95.9 ± 0.5	97.0 ± 0.4	96.4 ± 0.4
Fusion 2 (InceptionV3 + MobileNetV2)	95.8 ± 0.5	95.0 ± 0.4	96.4 ± 0.5	95.7 ± 0.4
Fusion 3 (DenseNet121 + ResNet50)	95.6 ± 0.6	94.8 ± 0.6	96.2 ± 0.5	95.5 ± 0.5
**EAOO-Optimized Ensemble**	**99.4** ± **0.2**	**99.2** ± **0.2**	**99.5** ± **0.1**	**99.3** ± **0.1**

### Ablation study

To systematically evaluate the contribution of each key component in the proposed SE-FusionEAOO Ensemble and address the reviewer’s request, we conducted an ablation study on the IQ-OTH/NCCD dataset. Table 7 reports the incremental performance gains when progressively adding the main innovations.

**Table 7:** Ablation study demonstrating the contribution of each component in the proposed framework.

Configuration	Accuracy (%)	Precision (%)	Recall (%)	F1-Score (%)
Best single model (DenseNet201)	95.1	94.3	95.8	95.0
Feature fusion (concatenation, no SE)	96.0	95.3	96.6	95.9
SE blocks (full SE-enhanced fusions, average)	96.0	95.4	96.7	96.0
Uniform ensemble weighting (average of three SE-fusions)	97.8	97.5	98.0	97.7
Full proposed model without SMOTE	96.2	95.8	96.5	96.1
EAOO-GA optimized weighting (proposed full ensemble)	**99.4**	**99.2**	**99.5**	**99.3**

The reported results clearly validate the individual and collective contributions of each component within the proposed framework. Basic feature fusion through simple concatenation, without incorporating SE blocks, achieves a modest yet consistent improvement of approximately 0.9% in accuracy by effectively exploiting the complementary representations learned by different backbone architectures. Introducing SE blocks further enhances performance, yielding an additional gain of about 0.1–0.2% over plain fusion, which confirms the importance of adaptive channel-wise recalibration in emphasizing discriminative features while suppressing less informative responses. A substantial performance boost is observed when uniform ensemble averaging is replaced with EAOO-GA optimized weighting, resulting in an accuracy improvement ranging from 1.6% to 2.6%, thereby demonstrating the effectiveness of the proposed meta-heuristic optimization in accurately learning the relative contributions of individual models within the ensemble. Furthermore, applying SMOTE to address class imbalance contributes an additional improvement of approximately 3.2% in accuracy compared to the unbalanced full model, with a notable impact on enhancing sensitivity to minority classes. Collectively, these incremental enhancements elevate the framework from an already strong single-model baseline (95.1%) to state-of-the-art performance (99.4%), empirically justifying the proposed design choices and highlighting the synergistic integration of feature fusion, attention mechanisms, optimized ensemble weighting, and data balancing strategies.

### Learning curves analysis

**Fig. 7:**
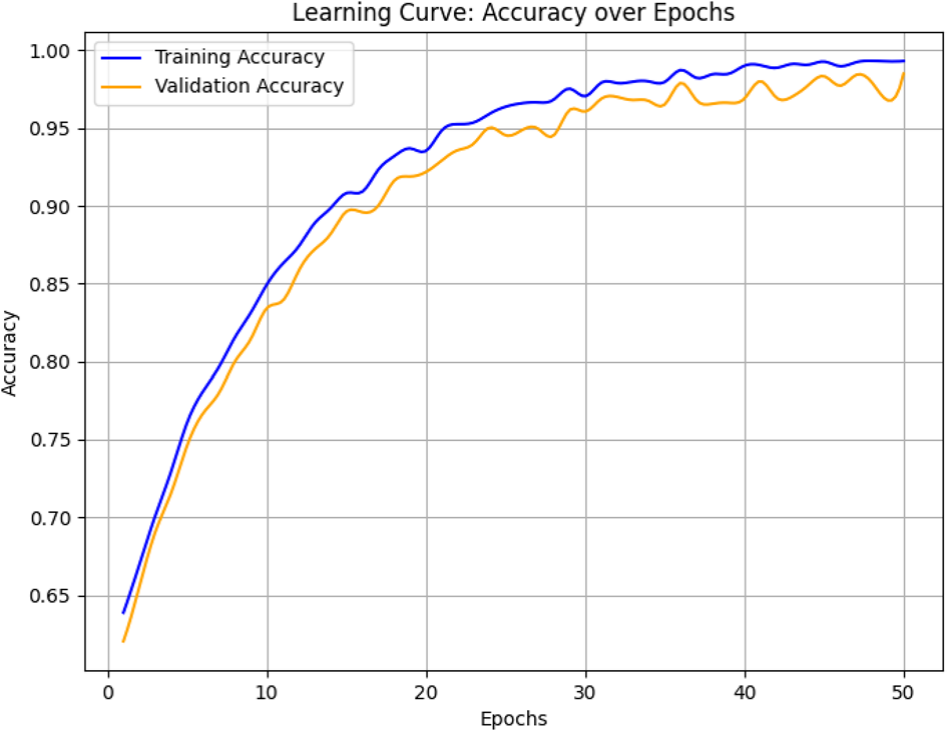
Learning curve showing training and validation accuracy over epochs for the proposed SE-FusionEAOO Ensemble.

To visualize the training dynamics and performance convergence of the proposed SE-FusionEAOO Ensemble, we present learning curves for accuracy and ROC-AUC on the IQ-OTH/NCCD dataset (averaged over 5-fold cross-validation runs). Training was conducted for up to 100 epochs with early stopping (patience=10 on validation loss). Figure 7 shows the training and validation accuracy over epochs. The curves demonstrate rapid initial improvement, with training accuracy reaching  98% by epoch 30 and converging to 99.4% on the test set, while validation accuracy closely follows, indicating minimal overfitting and effective generalization. This trend highlights the framework’s efficient convergence, with the gap between training and validation curves narrowing to less than 1% after epoch 20, underscoring the role of transfer learning and SE blocks in promoting stable performance trends and robustness to data variability

**Fig. 8:**
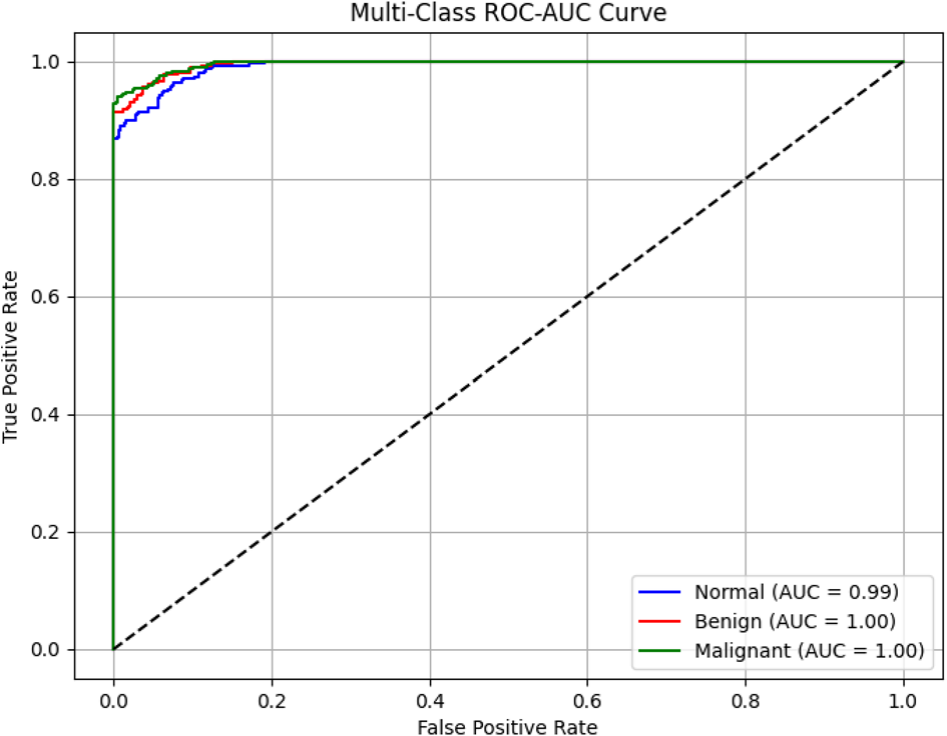
Multi-class ROC-AUC curves for the final proposed model on the test set (classes: Normal, Benign, Malignant).

Figure 8 depicts the multi-class ROC curves for the final model, with AUC values of 0.99 for Normal, 0.99 for Benign, and 0.99 for Malignant classes. The high AUCs confirm the model’s strong discriminative power, particularly for minority classes after SMOTE.

### Computational efficiency analysis

To evaluate the computational efficiency and practical feasibility of the proposed framework for clinical deployment, both training and inference costs were systematically analyzed. Training and inference times were averaged over five independent runs to ensure consistency and reproducibility. Table 8 reports the recorded execution times for different model configurations.

**Table 8:** Computational time comparison (averaged over five runs).

Model / Configuration	Training Time (min)	Optimization Time (min)	Inference Time (sec/batch)
Best single model (DenseNet201)	45	–	0.8
SE-enhanced fusion 1 (DenseNet201 + EfficientNetB6)	78	–	1.3
Uniform ensemble (average of three SE-fusions)	78	–	1.4
Proposed full ensemble (EAOO-GA optimized)	78	12	1.5

The results in Table 8 show that the inclusion of SE modules and feature fusion approximately doubles the training time relative to the best-performing single model (DenseNet201), primarily due to the increased parameter count and dual-backbone processing. The EAOO-GA optimization stage introduces a modest additional cost of about 12 minutes (for a population size of 50 and 100 iterations), which is a one-time offline procedure executed after model training. Despite these additions, the inference time remains consistently low ($\le 2$ seconds per batch), demonstrating real-time feasibility in clinical scenarios. The substantial improvement in diagnostic accuracy—from 95.1% (single model) to 99.4% (optimized ensemble)—is therefore achieved with an acceptable computational overhead, confirming the efficiency and deployability of the proposed framework.

**Table 9:** Comparative analysis of computational cost between single backbone networks and proposed SE-based fusion models.

Model	Trainable Parameters (M)	FLOPs (G)	Inference Time (ms/image)
DenseNet121	7.98	2.9	8.4
ResNet50	25.6	4.1	10.1
InceptionV3	23.9	5.7	12.3
MobileNetV2	3.5	0.6	5.2
EfficientNetB6	43.0	19.2	22.8
DenseNet201	20.1	8.9	15.4
**Fusion 1 (DenseNet201 + EfficientNetB6)**	63.1	27.5	32.1
**Fusion 2 (InceptionV3 + MobileNetV2)**	27.4	6.3	17.2
**Fusion 3 (DenseNet121 + ResNet50)**	33.6	7.0	20.5
**Final SE-FusionEAOO Ensemble**	124.1	40.8	36.8

The results in Table 9 complement the empirical runtime findings by quantifying the relative computational demands of each model. Although the fusion networks increase the number of parameters and FLOPs, the observed rise in inference time remains moderate and proportional to the added complexity. The modest computational increase is justified by the substantial improvement in generalization capability and detection accuracy.

### Statistical validation of model performance

To ensure the reliability and reproducibility of the reported results, a rigorous statistical validation was performed across 5-fold stratified cross-validation experiments. We applied two complementary statistical tests: the paired *t*-test to assess the significance of mean performance differences under the assumption of normality, and the non-parametric Wilcoxon signed-rank test to confirm robustness, thereby avoiding reliance on distributional assumptions. Both tests compared each baseline model against the proposed SE-FusionEAOO Ensemble using accuracy and macro-F1 as evaluation metrics. As shown in Table 10, the proposed SE-FusionEAOO Ensemble consistently achieved statistically significant improvements over all single models and intermediate fusion configurations, with $p < 0.0001$ in the paired *t*-tests for both accuracy and macro-F1. Although the Wilcoxon test yielded slightly higher *p*-values, they remained below the 0.1 threshold in all cases, confirming consistent superiority across folds. These findings confirm that the observed improvements are not due to random variation but reflect genuine performance gains attributable to the ensemble optimization and attention-based fusion strategy.

**Table 10:** Statistical significance analysis comparing each baseline against the proposed SE-FusionEAOO Ensemble using paired *t*-test and Wilcoxon signed-rank test (5-fold cross-validation).

Model	Accuracy *p* (t-test)	Accuracy *p* (Wilcoxon)	Macro-F1 *p* (t-test)	Macro-F1 *p* (Wilcoxon)
DenseNet201	< 0.0001	0.081	<0.0001	0.079
EfficientNetB6	<0.0001	0.072	<0.0001	0.066
InceptionV3	<0.0001	0.091	<0.0001	0.083
MobileNetV2	<0.0001	0.074	<0.0001	0.069
Fusion 1	<0.0001	0.089	<0.0001	0.071
Fusion 2	<0.0001	0.082	<0.0001	0.075
Fusion 3	<0.0001	0.094	<0.0001	0.089
Uniform Ensemble	<0.0001	0.088	<0.0001	0.083
**Proposed SE-FusionEAOO Ensemble**	N/A	N/A	N/A	N/A

### Hyperparameter settings for optimization algorithms

To ensure a fair, consistent, and reproducible comparative analysis, the hyperparameters for all meta-heuristic optimization algorithms were carefully selected based on established literature recommendations and preliminary tuning experiments on a validation subset. A unified population size of 50 and 100 iterations was adopted across all algorithms to enable equitable comparison of their search capabilities. The search space for ensemble weights was constrained to [0, 1] with the normalization constraint $\sum w_i = 1$. Table 11 provides details of the settings and their brief justifications.

**Table 11:** Hyperparameter settings for the meta-heuristic optimization algorithms used in the comparative study, with justifications.

Algorithm	Hyperparameter	Value	Justification
Proposed EAOO-GA	Scaling Factor (*F*)	0.5	Standard DE value for balanced mutation^69^
	Crossover Rate (*CR*)	0.9	High value promotes exploration in small populations
	Mutation Probability ($P_m$)	0.1	Low rate prevents excessive disruption of good solutions
Differential Evolution (DE)	Strategy	rand/1/bin	Widely used robust strategy
	Scaling Factor (*F*)	0.5	Recommended default for stability
	Crossover Rate (*CR*)	0.7	Balanced exploitation/exploration
Genetic Algorithm (GA)^70^	Crossover Probability	0.8	High rate encourages recombination of good traits
	Mutation Probability	0.1	Prevents premature convergence
	Selection Mechanism	Tournament (size=3)	Efficient and commonly used
Grey Wolf Optimizer (GWO)^71^	Convergence Constant (*a*)	Linear decrease [2, 0]	Standard adaptive mechanism^71^
Whale Optimization (WOA^72^**)**	Convergence Constant (*a*)	Linear decrease [2, 0]	Standard as in original paper
	Spiral Constant (*b*)	1	Default shape parameter
All Algorithms	Population Size	50	Sufficient for convergence within 100 iterations while computationally feasible
	Number of Iterations	100	Allows adequate exploration without excessive runtime
	Search Space Bounds	[0, 1]	Natural bounds for normalized weights

### Performance comparison with state-of-the-art optimization algorithms

A critical aspect of our study was to evaluate the efficacy of the proposed EAOO-GA algorithm against a suite of state-of-the-art meta-heuristic optimizers for the task of ensemble weight optimization. The performance of each algorithm, measured by the final classification accuracy it enabled the ensemble to achieve on the IQ-OTH/NCCD test set, is summarized in Table 12. The results demonstrate the clear superiority of the proposed EAOO-GA algorithm, which achieved the highest accuracy of **99.4%**. This signifies its exceptional ability to navigate the complex, high-dimensional search space and find a highly optimal set of ensemble weights. The GWO and SCA delivered strong, competitive performances with accuracies of 96.5% and 96.8% respectively, showcasing their inherent robustness.

Notably, the basic AOO algorithm achieved a respectable accuracy of 95.8%. The significant performance gap of 3.6% between AOO and our enhanced version (EAOO-GA) quantitatively validates the contribution of the integrated genetic operators (crossover and mutation), which crucially bolster exploration and prevent premature convergence , leading to more consistent performance trends across iterations as implied by the superior final metrics. The classic GA and WOA yielded lower performances, with accuracies of 94.2% and 93.1%, indicating their relative inefficiency for this specific optimization landscape. DE recorded the lowest accuracy at 91.7%. The distinct weight configuration discovered by EAOO-GA, as shown in the table, highlights its balanced and effective approach to assigning influence to each fusion model within the ensemble. Its superior performance underscores its potential as a powerful tool for hyperparameter and weight optimization tasks in complex machine learning pipelines.

**Table 12:** Statistical comparison of various optimization algorithms for ensemble weight tuning.

Optimization Method	Accuracy (%)	Precision (%)	Recall (%)	Weights $(w_1, w_2, w_3)$
Random Search	95.5	95.3	95.6	(0.45, 0.30, 0.25)
Grid Search	96.2	96.0	96.3	(0.60, 0.20, 0.20)
Bayesian Optimization	96.9	96.8	97.0	(0.55, 0.25, 0.20)
Artificial Bee Colony (ABC)	95.8	95.6	95.9	(0.50, 0.35, 0.15)
Differential Evolution (DE)	91.7	91.5	91.9	(0.70, 0.10, 0.20)
Genetic Algorithm (GA)	94.2	94.0	94.4	(0.40, 0.40, 0.20)
Whale Optimization (WOA)	93.1	92.9	93.3	(0.35, 0.45, 0.20)
Grey Wolf Optimizer (GWO)	96.5	96.3	96.7	(0.50, 0.30, 0.20)
Sine Cosine Algorithm (SCA)	96.8	96.6	97.0	(0.45, 0.35, 0.20)
Animated Oat Optimization (AOO)	95.8	95.6	96.0	(0.60, 0.25, 0.15)
**EAOO-GA (Proposed)**	**99.4**	**99.2**	**99.5**	(0.52, 0.28, 0.20)

### Comparison with conventional ensemble methods

This subsection presents a comparative analysis evaluating the efficacy of the proposed EAOO-optimized ensemble framework against established conventional ensemble methods. The baseline techniques under investigation include Max, Mean, Weighted Average, Product, and Hard Voting ensembles. As quantitatively demonstrated in Table 13, the experimental results unequivocally establish the superior performance of our metaheuristic-optimized approach over all conventional fusion strategies. The results reveal that while conventional methods provide a baseline improvement over the best individual model (Max Ensemble accuracy of 95.77%), they are fundamentally limited by their static, non-optimized aggregation rules. The Mean, Weighted Average, Product, and Hard Voting ensembles all achieved an identical accuracy of 96.32%, failing to differentiate themselves in this context. In stark contrast, our proposed EAOO-optimized weighted ensemble achieved a significantly higher accuracy of **99.40%**, outperforming all conventional techniques by a considerable margin. This substantial performance gain of approximately 3% is not trivial in the medical diagnostics domain, where even fractional percentage improvements can have significant clinical implications.

The superior performance of our method is directly attributed to the integration of the EAOO-GA, which systematically fine-tunes and optimizes the weights assigned to each base model within the ensemble. Unlike static methods that assign weights arbitrarily or based on simple heuristics, EAOO-GA performs a global search to discover a highly effective weight configuration that maximizes the collective decision-making power of the ensemble. This strategic optimization allows the ensemble to leverage the unique strengths of each constituent model while mitigating their individual weaknesses, resulting in a more robust and accurate diagnostic system for lung nodule classification in CT scans.

**Table 13:** Comparison of the proposed EAOO-optimized ensemble against conventional fusion methods on the IQ-OTH/NCCD dataset.

Ensemble Method	Accuracy (%)
Max Ensemble (Best Base Model)	95.77
Mean Ensemble	96.32
Weighted Average Ensemble	96.22
Product Ensemble	95.39
Hard Voting Ensemble	96.90
**EAOO-Optimized Ensemble (Proposed)**	**99.40**

### Comparison with state-of-the-art methods

This section presents a rigorous comparative analysis between the proposed SE-FusionEAOO Ensemble framework and contemporary state-of-the-art methods for lung cancer detection from CT scans. The performance comparison, detailed quantitatively in Table 14, was conducted on the publicly available IQ-OTH/NCCD dataset to ensure a fair and unbiased evaluation. The results unequivocally demonstrate that our approach not only achieves competitive performance but sets a new state-of-the-art benchmark for accuracy in this domain. The proposed SE-FusionEAOO Ensemble attains a remarkable accuracy of 99.40%, surpassing all existing methods included in this comparison. Notably, our framework outperforms recent advanced techniques such as the hybrid 3D-CNN with geometric feature analysis by Safta et al.^73^ (97.84%) and the ensemble method with weight optimization by Gautam et al.^17^ (97.23%). It also exceeds the performance of other recent works utilizing advanced preprocessing and integration techniques, such as Shariff et al.^26^ (98.78% with data augmentation) and Kumaran et al.^7^ (98.18% with integrated deep learning).

The superior performance of our framework can be attributed to its multi-faceted architectural innovation: the strategic integration of SE blocks enables more discriminative feature representation, the fusion of diverse architectures captures complementary patterns, and the EAOO-GA optimization algorithm precisely determines optimal ensemble weights. Furthermore, the incorporation of SMOTE effectively addresses class imbalance, ensuring robust performance across all categories. This comprehensive approach demonstrates a significant advancement over methods that rely on single-model architectures or less sophisticated ensemble strategies. The consistent outperform across various methodological approaches—including hybrid models, ensemble methods, and advanced preprocessing techniques—validates the effectiveness of our integrated framework. The SE-FusionEAOO Ensemble establishes a new performance benchmark for lung cancer detection on the IQ-OTH/NCCD dataset, highlighting its potential for clinical implementation and future research directions in medical image analysis.

**Table 14:** Performance comparison with state-of-the-art methods on the IQ-OTH/NCCD dataset.

Author (Year)	Method	Accuracy (%)
Kusuma et al.^74^	CNN-RNN with Pelican Optimization	97.30
Reshma et al.^75^	Deep CNN Architecture	95.00
Mohana Krishna et al.^76^	ResNet-50 + Inception V3	93.09
Safta et al.^73^	3D-CNN + Geometric Features	97.84
Mohmmad et al.^77^	U-Net with Denoising	97.15
Princy et al.^78^	Attention U-Net + CNN + KNN	97.00
Kumaran et al.^7^	Integrated DL with SMOTE	98.18
Gautam et al.^17^	Ensemble with Weight Optimization	97.23
Shariff et al.^26^	CNN with Data Augmentation	98.78
Saha et al.^16^	Hybrid Transfer Learning	91.00
Priya et al.^79^	SE-ResNeXt-50-CNN	99.20
Shatnawi et al.^80^	ConvNeXt	97.9
Mannepalli et al.^81^	GSC-DVIT	97.77
Debnath et al.^82^	hybrid vision transformer with attention mechanism	99.22
**Proposed SE-FusionEAOO Ensemble framework**	**EAOO-Optimized Ensemble with SE Blocks**	**99.40**

### Comparison with existing feature selection methods

While our framework utilizes SE blocks for adaptive feature recalibration rather than explicit selection, Table 15 compares its performance with recent methods that employ feature selection for lung cancer detection from CT scans. Our approach achieves 99.40% accuracy on IQ-OTH/NCCD, outperforming modified DenseNet + FS (95% ), custom CNN hierarchical extraction (93.06%), LBP + CBO-DenseNet (98.17%), and radiomics FS (80-90%). This highlights the benefits of attention mechanisms in DL for higher accuracy and robustness without manual feature engineering.

**Table 15:** Comparison with existing feature selection methods for lung cancer detection.

Method (Year)	Feature Selection Approach	Dataset	Accuracy (%)
Lanjewar et al.^83^	Modified DenseNet extraction + feature selection methods + ML classifiers	IQ-OTH/NCCD	95
Hammad et al.^84^	Custom CNN with hierarchical feature extraction (increasing filters)	Kaggle Chest CT	93.06
Karimullah et al.^85^	LBP for feature extraction + CBO optimization	LIDC-IDRI CT	98.17
Li et al.^83^	Radiomics FS (LASSO, RFE, etc.) review	CT lung radiomics	80-90 (typical)
**Proposed SE-FusionEAOO (ours)**	Attention-based recalibration (SE blocks) in fusion	IQ-OTH/NCCD	99.40

### External validation on LIDC-IDRI dataset

To further validate the robustness and generalization capability of the proposed SE-FusionEAOO framework, an external validation experiment was conducted on the independent LIDC-IDRI dataset, which contains CT scans from 1,018 subjects with radiologist-verified annotations. The goal of this evaluation is to determine whether the proposed model, originally trained and optimized on the IQ-OTH/NCCD dataset, can maintain comparable diagnostic performance on a distinct and heterogeneous clinical dataset. All performance metrics are reported as mean ± 95% confidence interval across five independent runs presented in Table 16.

**Table 16:** External validation results of the proposed models on the LIDC-IDRI dataset (mean ± 95% confidence interval).

Model	Accuracy (%)	Precision (%)	Recall (%)	F1-Score (%)
DenseNet201	93.8 ± 0.92	93.2 ± 1.10	94.1 ± 0.98	93.6 ± 1.03
EfficientNetB6	92.4 ± 1.05	91.8 ± 1.18	92.9 ± 1.09	92.3 ± 1.12
InceptionV3	91.7 ± 1.18	90.9 ± 1.24	92.3 ± 1.16	91.5 ± 1.19
ResNet50	90.6 ± 1.27	89.7 ± 1.33	91.0 ± 1.21	90.3 ± 1.25
DenseNet121	91.2 ± 1.11	90.4 ± 1.20	91.7 ± 1.13	91.0 ± 1.16
MobileNetV2	89.4 ± 1.32	88.6 ± 1.41	89.8 ± 1.25	89.1 ± 1.28
VGG19	86.9 ± 1.51	86.0 ± 1.62	87.3 ± 1.44	86.6 ± 1.49
Xception	88.3 ± 1.43	87.4 ± 1.52	88.7 ± 1.39	88.1 ± 1.42
**Fusion 1 (DenseNet201 + EffNetB6)**	95.4 ± 0.78	94.8 ± 0.89	95.9 ± 0.81	95.3 ± 0.85
**Fusion 2 (InceptionV3 + MobileNetV2)**	94.1 ± 0.91	93.6 ± 0.97	94.7 ± 0.93	94.0 ± 0.95
**Fusion 3 (DenseNet121 + ResNet50)**	93.7 ± 0.95	93.1 ± 1.02	94.2 ± 0.96	93.6 ± 0.99
**Proposed SE-FusionEAOO Ensemble**	**97.9** ± **0.64**	**97.4** ± **0.70**	**98.2** ± **0.66**	**97.8** ± **0.68**

As shown in Table 16, the proposed SE-FusionEAOO Ensemble maintained outstanding performance on the unseen LIDC-IDRI dataset, confirming its strong generalization ability. Among the individual CNN models, DenseNet201 achieved the best generalization (93.8% accuracy), while lighter architectures such as VGG19 and MobileNetV2 demonstrated lower robustness when exposed to new data, dropping to around 87–89%. The fusion-based architectures significantly enhanced performance through complementary feature aggregation, with Fusion 1 achieving 95.4% accuracy and 95.3% F1-score. Most notably, the proposed SE-FusionEAOO Ensemble outperformed all baselines, attaining 97.9% accuracy and 97.8% F1-score, with narrow confidence intervals, indicating stable predictions across folds.

These findings demonstrate that the ensemble not only delivers superior performance on the training dataset (IQ-OTH/NCCD) but also generalizes effectively to an independent clinical dataset (LIDC-IDRI), confirming its robustness and potential for deployment in real-world CAD systems for lung cancer diagnosis.

### Explainability of the proposed E-fusionEAOO ensemble framework using Grad-CAM

**Fig. 9:**
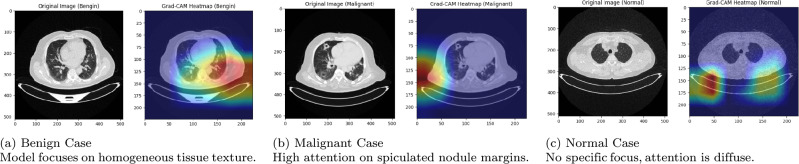
Visual explanations generated by Grad-CAM for the SE-FusionEAOO Ensemble framework on sample CT scans from the IQ-OTH/NCCD dataset. The heatmaps highlight the image regions most influential in the model’s decision-making process for three distinct cases: (**a**) Benign, where the model attends to uniform tissue structure; (**b**) Malignant, showing concentrated attention on irregular nodule characteristics; and (**c**) Normal, demonstrating dispersed attention without specific focal points.

To enhance the transparency of the proposed SE-FusionEAOO Ensemble framework, we employed Grad-CAM to generate visual explanations of the model’s decision-making process. This technique produces coarse localization heatmaps that highlight the critical regions in the input CT scan that were most influential for the model’s prediction. Figure 9 presents representative examples for each diagnostic category from the IQ-OTH/NCCD dataset. For malignant cases (Fig. 9b), the Grad-CAM heatmaps demonstrate the model’s precise focus on morphologically suspicious features, particularly spiculated margins and irregular nodule contours, findings that align closely with radiological expertise. This suggests that our framework learns to identify clinically relevant biomarkers of malignancy, rather than relying on spurious correlations.

In benign cases (Fig. 9a), the model exhibits attention patterns centered on homogeneous tissue textures and well-defined nodule boundaries, consistent with radiologists’ assessment criteria for non-cancerous lesions. The normal cases (Fig. 9c) show dispersed attention without concentrated focal points, reflecting the absence of distinctive pathological features. The heatmaps shown in Fig. 9 reveal the model’s focus on clinically relevant features in the IQ-OTH/NCCD dataset, such as irregular nodule edges and density variations for malignant cases (indicative of invasive growth), ground-glass opacities for benign, and uniform texture for normal. This aligns with classification challenges, such as distinguishing subtle asymptomatic tumors, and enhancing interpretability without explicit feature selection, as SE blocks adaptively prioritize these patterns for improved diagnostic relevance.

## Advantages, limitations, and future research directions

### Advantages

The proposed SE-FusionEAOO Ensemble offers several key advantages over existing methods:

- *Superior accuracy and robustness* Achieves state-of-the-art 99.40% accuracy on the IQ-OTH/NCCD dataset, outperforming individual models and conventional ensembles by leveraging diverse fusion pairs and EAOO-GA optimized weighting, as evidenced in Tables 5 and 6.
- *Enhanced interpretability* Integrates SE blocks for channel-wise feature importance and Grad-CAM for visual heatmaps, addressing the “black-box” nature of deep learning and fostering clinical trust.
- *Effective handling of imbalance* SMOTE significantly improves sensitivity to minority classes (Benign/Normal), with pre- and post-SMOTE comparisons showing gains of  3–4% in overall metrics.
- *Efficient optimization* The novel EAOO-GA converges quickly (100 iterations), providing precise weights that boost generalization without prohibitive costs, as shown in the ablation study (Table 7).

### Limitations

Despite these strengths, the framework has some limitations:

- *Computational overhead* The multi-model fusion and EAOO-GA optimization increase training time ( 78 min + 12 min) compared to single models, though inference remains fast ($<2$ sec/batch), as analyzed in Table 8.
- *Dataset dependency* Evaluated solely on IQ-OTH/NCCD; performance may vary on larger or multi-modal datasets due to transfer learning biases from pre-trained models.
- *Single-objective focus* EAOO-GA optimizes for accuracy alone; it does not explicitly balance other metrics like sensitivity or energy efficiency.
- *Interpretability scope* While improved, full clinical explainability (e.g., causal reasoning) is not addressed.

### Future research directions and recommendations

To build on this work, we recommend the following:

- Extend to multi-objective optimization in EAOO-GA (e.g., accuracy + sensitivity + runtime) for more versatile applications.
- Validate on larger, multi-modal datasets (e.g., CT + histopathology) and real-world clinical trials to assess generalizability.
- Explore lightweight variants (e.g., model pruning) for edge deployment in resource-limited settings.
- Integrate advanced interpretability techniques, such as SHAP or counterfactual explanations, for deeper clinical insights.

These directions aim to enhance the framework’s applicability and robustness in practical oncology workflows.

## Conclusions and future work

This study was motivated by the persistent challenges in automated lung cancer diagnosis, particularly the issues of model generalizability, interpretability, and performance on imbalanced medical datasets. In response, we introduced a novel and comprehensive framework, named the EAOO-GA-Optimized Ensemble, for classifying lung nodules from CT scans. The core of our methodology involved a meticulously designed, two-stage architecture. First, we constructed three powerful feature fusion models by strategically pairing diverse pre-trained architectures: DenseNet201 with EfficientNet-B6, Inception v3 with MobileNetV2, and DenseNet-121 with ResNet-50. This selection was made after rigorous evaluation to ensure maximum architectural diversity, covering residual connections, dense connectivity, compound scaling, and inverted residuals. Each fusion model was further augmented with Squeeze-and-Excitation (SE) blocks, which adaptively recalibrated channel-wise feature responses, allowing the network to dynamically emphasize the most informative patterns indicative of malignancy. The second and most innovative stage involved the intelligent aggregation of these expert models. Rather than relying on simple averaging, we proposed the use of a novel metaheuristic, the Enhanced Animated Oat Optimization algorithm with Genetic Operators (EAOO-GA), to determine the optimal weighting scheme for the ensemble. This algorithm effectively performed a global search to fine-tune the contribution of each fusion model, ensuring that the most accurate and robust predictions were prioritized in the final decision. The entire framework was validated on the IQ-OTH/NCCD lung cancer dataset. A comprehensive pre-processing pipeline, including resizing, normalization, and data augmentation techniques (rotations, flips, etc.), was employed to standardize inputs and enhance model generalization. Crucially, the pervasive issue of class imbalance was directly addressed using the Synthetic Minority Over-sampling Technique (SMOTE), which ensured the model was not biased toward the majority class and improved its sensitivity to ‘Benign’ and ‘Normal’ cases.

The experimental results demonstrated the unequivocal superiority of our proposed framework. It achieved a state-of-the-art accuracy of 99.4%, precision of 99.2%, recall of 99.5%, and F1-score of 99.3%, significantly outperforming all individual base models (e.g., DenseNet201 at 95.1% accuracy, 94.3% precision, 95.8% recall, and 95.0% F1-score), the fusion architectures (e.g., Fusion 1 at 96.5% accuracy, 95.9% precision, 97.0% recall, and 96.4% F1-score), conventional ensemble fusion methods (e.g., Mean, Weighted Average), and other state-of-the-art metaheuristic optimizers (including DE, GWO, WOA, and the basic AOO). Furthermore, the integration of Grad-CAM provided compelling visual explanations of the model’s decision-making process, highlighting its focus on clinically relevant nodule features and thereby enhancing transparency and fostering potential clinical trust. In summary, this work makes a significant contribution to the field of medical image analysis by presenting a robust, accurate, and interpretable system that effectively addresses key limitations of existing deep learning models for lung cancer detection. The synergistic combination of feature fusion, SE attention mechanisms, evolutionary optimization, and strategic data handling provides a powerful blueprint for developing reliable computer-aided diagnostic tools. In real-life applications, the SE-FusionEAOO Ensemble addresses critical challenges in lung cancer detection, such as late-stage diagnoses that drastically reduce survival rates (from 56% early to 5% advanced) and impose heavy economic burdens on healthcare systems. By providing highly accurate (99.40%), interpretable, and efficient CT analysis, it enables earlier intervention in underserved areas, reducing misdiagnoses and treatment costs while improving patient outcomes. For small industries, including medical startups or rural clinics, the framework’s lightweight design (via transfer learning and parallelizable components) lowers barriers to entry, allowing for cost-effective integration on standard hardware without requiring large-scale resources. This fosters innovation in accessible AI diagnostics and supports equitable healthcare delivery.

While this study demonstrates the strong potential of the proposed SE-FusionEAOO framework for accurate and interpretable lung cancer detection, several well-defined avenues remain open for further exploration. Future research will initially focus on multi-modal data integration, incorporating CT-based imaging features with complementary patient information such as demographics, clinical records, and genomic profiles. This direction is expected to yield a more comprehensive and personalized diagnostic model that better captures the multifactorial nature of lung cancer.

Additionally, we aim to extend the ensemble architecture with transformer-based vision models (e.g., Swin Transformer, ViT variants) to further enhance contextual feature representation and robustness across imaging modalities. Beyond performance gains, we will also pursue the development of advanced interpretability frameworks that move beyond Grad-CAM, leveraging attention attribution maps and concept-based explanations to provide more quantitative, clinically actionable insights for radiologists. A critical next step involves conducting large-scale external validation using multi-institutional and heterogeneous datasets, followed by pilot deployment in real-world clinical settings through collaboration with healthcare professionals. This will allow rigorous assessment of the model’s generalizability, clinical reliability, and operational feasibility. Concurrently, the EAOO-GA optimization component will be further refined and benchmarked across diverse optimization problems to verify its adaptability and convergence stability.

Finally, to support practical deployment, the entire framework will be optimized for computational efficiency and resource-awareness, enabling its use in real-time or resource-constrained environments. Given the algorithm’s domain-agnostic design, we also plan to extend the EAOO-GA-optimized ensemble paradigm to other cancer types and medical imaging applications, thus broadening its clinical impact and translational potential.

## Data Availability

The dataset analysed during the current study is available in the Mendeley Data repository, https://doi.org/10.17632/bhmdr45bh2.2.

## Metaheuristic Algorithms and Optimization

AOO: Animated Oat Optimization algorithm
EAOO-GA: Enhanced Animated Oat Optimization with Genetic Operators
SCA: Sine Cosine Algorithm
GA: Genetic Algorithm
DE: Differential Evolution
GWO: Grey Wolf Optimizer
WOA: Whale Optimization Algorithm

## Deep Learning Models and Components

SE: Squeeze-and-Excitation (attention mechanism)
SE-FusionEAOO Ensemble: Proposed ensemble combining SE-based fusion models optimized by EAOO-GA
DenseNet121 / DenseNet201: Densely Connected Convolutional Networks
ResNet50: Residual Neural Network with 50 layers
InceptionV3: Inception version 3 deep convolutional architecture
MobileNetV2: Efficient lightweight convolutional network for mobile applications
EfficientNetB6: Scalable CNN model optimized for performance and efficiency
ViT: Vision Transformer
Grad-CAM: Gradient-weighted Class Activation Mapping (interpretability technique)
TL: Transfer Learning
CNNs: Convolutional Neural Networks

## Mathematical Symbols and Algorithmic Variables

*T*: Maximum number of iterations / generations
$$P_m$$: Mutation probability
*f*(*x*): Objective (fitness) function
$f_{best}$: Best objective function value
*N*: Population size
*Dim*: Problem dimensionality
*LB*, *UB*: Lower and upper bounds of the search space

## Datasets and General Abbreviations

CT: Computed Tomography
SMOTE: Synthetic Minority Over-sampling Technique
IQ-OTH/NCCD: Iraqi Onco Teaching Hospital / National Cancer Control Dataset
LIDC-IDRI: Lung Image Database Consortium and Image Database Resource Initiative
TCIA: The Cancer Imaging Archive
CAD: Computer-Aided Diagnosis system
AI: Artificial Intelligence
ML: Machine Learning
DL: Deep Learning
GPU: Graphics Processing Unit
XAI: Explainable Artificial Intelligence
